# Wireless Energy Harvesting Two-Way Relay Networks with Hardware Impairments

**DOI:** 10.3390/s17112604

**Published:** 2017-11-13

**Authors:** Chunling Peng, Fangwei Li, Huaping Liu

**Affiliations:** 1 Chongqing Key Lab of Mobile Communications Technology, Chongqing University of Posts and Telecommunications, Chongqing 400065, China; chunlingp@163.com; 2 School of Electrical Engineering and Computer Science, Oregon State University, Corvallis, OR 97331, USA; huaping.liu@oregonstate.edu

**Keywords:** energy harvesting, two-way relay, hardware impairments, ergodic capacity, optimal power splitting

## Abstract

This paper considers a wireless energy harvesting two-way relay (TWR) network where the relay has energy-harvesting abilities and the effects of practical hardware impairments are taken into consideration. In particular, power splitting (PS) receiver is adopted at relay to harvests the power it needs for relaying the information between the source nodes from the signals transmitted by the source nodes, and hardware impairments is assumed suffered by each node. We analyze the effect of hardware impairments on both decode-and-forward (DF) relaying and amplify-and-forward (AF) relaying networks. By utilizing the obtained new expressions of signal-to-noise-plus-distortion ratios, the exact analytical expressions of the achievable sum rate and ergodic capacities for both DF and AF relaying protocols are derived. Additionally, the optimal power splitting (OPS) ratio that maximizes the instantaneous achievable sum rate is formulated and solved for both protocols. The performances of DF and AF protocols are evaluated via numerical results, which also show the effects of various network parameters on the system performance and on the OPS ratio design.

## 1. Introduction

Energy harvesting (EH) has recently attracted enormous attention from researchers as a promising supplemental technology in prolonging the lifetime of a wireless network, especially in wireless sensor networks [1], cognitive radio networks [2], etc. With energy harvesting technique, the energy can be captured through solar power [3], strongly coupled magnetic resonances [4] or radio-frequency (RF) signals in ambient environments [5]. Since RF signals carry energy as well as information, the integration of RF energy harvesting capabilities into wireless communication systems provides a possibility of simultaneous wireless information and power transfer (SWIPT) [6]. The pioneer work of initial work on SWIPT is discussed in [7], which considers an ideal receiver design that is able to simultaneously observe and extract power from the same received signal. However, as discussed in [8], this such an ideal receiver does not hold exist in practice. For practical implementation, the study in [8] also introduces two realizable receiver architectures design: time switching (TS) and power splitting (PS).

Cooperative relay network [9,10] is a common application scenario of SWIPT. In relay networks, the relay expenditures additional resources for relaying, which might result a decreased lifetime for energy-constrained relay networks. A handful of research efforts have explored SWIPT for one-way relay (OWR) and two-way relay (TWR) networks. In [11], Nasir et al. analyze TS and PS protocols for one-way amplify-and-forward (AF) relay networks, and derive analytical expressions for the outage probability and the ergodic capacity. Yin et al. [12] study the optimal power splitting design to maximize the cooperative capacity of OWR networks with AF and decode-and-forward (DF) protocols. The work in [13] investigates the impact of SWIPT on the performance of one source-destination, multiple-relay cooperative networks and derive a closed-form expression of the outage probability. With relay selection, the a closed-form expression of the outage probability has been derived and verified over independent Nakagami-*m* fading channels in [14] for a dual-hop wireless powered cooperative system. For two-way relaying AF networks with the PS protocol, Chen et al. [15] analytically derive the exact expressions, tight upper and lower bounds on the outage probability and the ergodic capacity. In [16], resource allocation of the PS and time phases ratios for the PS-based SWIPT and that of the TS and time phases ratios for the TS-based SWIPT are studied to minimize the system outage probability in TWR networks.

The above research works contribute in the area of assuming that the transceiver and receiver hardware of all nodes is perfect. In practice, however, the hardware of a wireless node undergo several kinds of impairments such as phase noise, I/Q imbalance, high power amplifier non-linearities, etc. [17,18]. There are several works have investigated the effect of hardware impairments (HI) in relay networks, which are illustrated next. In [19], the authors quantify the impact of HI on dual-hop AF and DF relaying networks and derive the outage probability subject to Nakagami-*m* fading. Matthaiou et al. [20] analyze the impact of HI at the relay in an AF-TWR configuration, and deduce a closed-form expression for the outage probability (OP) and symbol-error rates (SERs). In [21], the impact of HI on spectrum underlay cognitive multiple-relay networks is studied. You et al. [22] study the joint source/relay precoding design for a practical MIMO two-way AF relaying system that suffers from HI. Recently, there emerges a few recent works concentrating on analyzing analyzed the impact of HI on energy harvesting enabled relay networks. Do et al. [23] consider the joint impacts of EH and HI on multiple-relay one-way networks, and analyze the OP under two relay selection protocols. In [24], the outage probability and throughput are investigated for cognitive two-way DF relay networks, under the effects of realistic relay transceiver structures are taken into consideration. It is evident that the research work in [23,24] provides contributions on protocol design and outage performance analysis for energy harvesting DF relay networks with HI. However, Do, et al. [23] analyzed OWR networks, and ignores the multiple-access constraint in TWR with TS and PS protocols in [24]. Meanwhile, the ergodic capacity and optimal energy harvesting design under considering the impact of hardware impairments is lack of exploration.

In this paper, we focus on a two-way passive-relay network in which a power-splitting receiver is adopted at the relay to replenish energy under the consideration of hardware impairments at each node. The exact analytical expressions of the achievable sum rate and ergodic capacities in integral closed-form are derived for both DF and AF protocols and address the optimization problems allocated to energy harvesting and for both protocols to maximize the achievable sumrate, respectively. The main contributions of this paper can be described in more details as follows.

- We have presented a self-powered TWR energy harvesting and signal transmission models for both DF and AF protocols suffered by hardware impairments considered at all nodes.
- We obtained the new signal-to-noise-plus-distortion ratio (SNDR) expressions, and derived the exact analytical expressions of the achievable sum rate and ergodic capacities in integral closed-form for both DF and AF protocols respectively.
- In order to obtain more engineering insights, we formulate and solve the optimal power splitting (OPS) ratio that maximizes the instantaneous achievable sum rate for both DF and AF protocols.
- Simulation and numerical results are presented to verify our derivation and to assess the effects of various parameter settings on system performance. The achieved sum rate with the OPS design are compared that with the equal power splitting (EPS) design, and the performance of DF and AF protocols are also compared and discussed.

The rest of this paper is organized as follows. In Section 2, we describes the system and signal models, and presents the new expressions of SNDRs and the instantaneous achievable sum rate expressions. The derivation of ergodic capacities of both DF and AF protocols are presented in Section 3. Section 4 formulates and develops solutions of OPS ratio for DF and AF protocols. Section 5 validates the analytical results and assesses the effects of various network parameter settings on the performance and optimal solutions via numerical results, followed by concluding remarks in Section 6.

## 2. System and Signal Model

**Figure 1:**
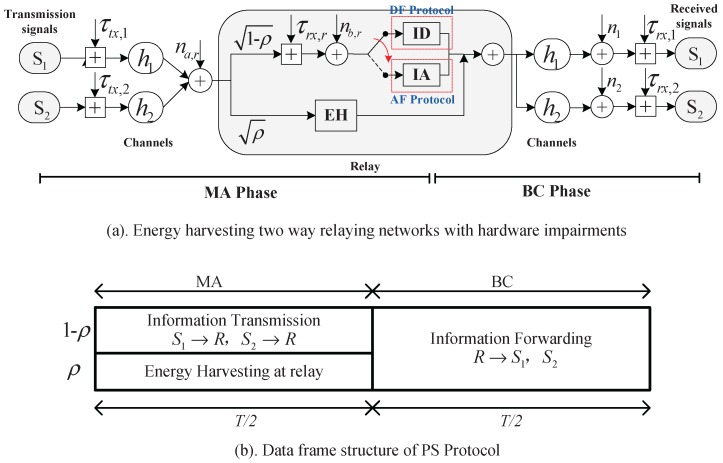
System model: (**a**) energy harvesting two way relaying networks with hardware impairments; (**b**) data frame structure of the PS protocol.

Consider a typical half-duplex two-way relay network that consists of three nodes: two source nodes $S_{1}$ and $S_{2}$ with continuously their power sources, and an energy harvesting relay node *R*, which harvests the energy it needs from the RF signals transmitted by the two source nodes. It is assumed that there are no direct links between the two source nodes due to deep shadowing or blockage; thus information exchange between them only relies on the relay. A PS receiver architecture is employed at the relay as an energy harvester, and hardware impairments that result in distortion noises suffered by all nodes are modeled as shown in Figure 1a.

### 2.1. TWR EH and Hardware-Impairment-Distortion Model

The network employs a two-step multiple access broadcast transmission and assumes two common relaying protocols, decode-and-forward (DF) protocol and amplify-and-forward (AF). In this configuration, each round of transmission stage consist of two phases: multiple access (MA) phase and broadcast (BC) phase. In the MA phase, the relay *R* harvests energy and collects receives data from the wireless signals transmitted by both source nodes simultaneously; in the BC phase, the relay processing the collected data it received through the DF protocol or the AF protocol, and then broadcasts the processed information $x_{R}$ with the harvested energy to both source nodes. Note that the power needed for information processing and for powering the relay hardware is ignored in the model. This kind of assumption has been applied in a lot of references, such as [11]).

The power splitting energy harvesting protocol is considered in this paper, in which the receiver architecture of the relay consists of a power splitter and an information decoder (ID) for the DF protocol is adopted, or an information amplifier (IA) for the AF protocol is adopted. The power splitter divides the received signal (transmitted by the source nodes) into two portions: one portion ρ(0≤ρ≤1) is used for EH and the other portion (1−ρ) for information processing.

Hardware impairments are considered both the transmitter and receiver of each node. Similar to [19,23], we model the received signal in the presence of hardware impairments for an end-to-end transmission as

(1) $$y=h\sqrt{P}\left(x+\tau_{tx}\right)+\tau_{rx}+n$$

where $\tau_{tx}∼\mathrm{CN}\left(0,\kappa_{tx}^{2}\right)$ and $\tau_{rx}∼\mathrm{CN}\left(0,\kappa_{rx}^{2}P_{\mathrm{rec}}\right)$ are the aggregate distortiona affecting noise caused by HI at the transmitter and receiver, respectively, $\kappa_{tx}$ and $\kappa_{rx}$ characterize the levels of impairments of the transmitter and receiver hardware, respectively, CN(·) stands for complex circularly symmetric Gaussian distributions, $P_{\mathrm{rec}}={\left|h\right|}^{2}P$ is power of the received signal expressed in (1), *h* is the channel coefficient and *n* is the additive white Gaussian noise. For simplicity of analysis, in the following discussion, we assume all nodes have the same structure so that the level of HI are the same, i.e., $\kappa_{tx,i}=\kappa_{tx,j}=\kappa_{1}$, $\kappa_{rx,i}=\kappa_{rx,j}=\kappa_{2}$, (i,j=1,2,r represent source $S_{1}$, source $S_{2}$ and relay *R* respectively).

### 2.2. Information Transmission in TWR

Information transmission in this paper consist of two phase, MA phase and BC phase. Let *T* indicate the duration of one round of transmission, from which half of the time, T/2, is used for MA phase, and the remaining half, T/2, is used for BC phase, which is shown in Figure 1b. In the MA phase, source nodes $S_{1}$ and $S_{2}$ transmit the information simultaneously.

With hardware impairments distortion mingled, the received signal at the relay can be written as

(2) $$y_{R}=h_{1}\sqrt{P_{1}}\left(x_{1}+\tau_{tx,1}\right)+h_{2}\sqrt{P_{2}}\left(x_{2}+\tau_{tx,2}\right)+\tau_{rx,r}+n_{r}$$

where $\tau_{tx,i}∼\mathrm{CN}\left(0,\kappa_{1}^{2}\right),i=1,2$, is the HI caused by the transmitter of $S_{i}$, $\tau_{rx,r}∼\mathrm{CN}(0,\kappa_{2}^{2}\left(\right|h_{1}{|}^{2}P_{1}+|h_{2}{|}^{2}P_{2}))$ is the HI caused by the receiver of *R*, $h_{i}$ denotes the channel coefficient between $S_{i}$ and *R*, $n_{r}=n_{a,r}+n_{b,r},n_{r}\;∼\;\mathrm{CN}\left(0,\sigma_{r}^{2}\right)$ is the additive white noise generated at the relay. And $n_{a,r}\;∼\;\mathrm{CN}\left(0,\sigma_{a}^{2}\right)$ is the noise generated at the receiver antenna, and $n_{b,r}\;∼\;\mathrm{CN}\left(0,\sigma_{b}^{2}\right)$ is the noise generated in the down-conversion process of the received signal to baseband [11]. Since in practice the power of the noise generated at the antenna ($\sigma_{a}^{2}$) is generally much smaller than the noise power introduced by the receiver chain ($\sigma_{b}^{2}$), for simplicity, we neglect the noise term $n_{a,r}$ and adopt $n_{r}=n_{b,r}$ in the following analysis.

Next, the relay then splits the received signal into two portions: $\sqrt{\rho }y_{R}$ is direct to the energy harvester for EH, $\sqrt{1-\rho }y_{R}$ is direct to the information processing (IP) circuit for information decoding (ID), if the relay works in DF protocol or information amplify (IA) if the relay works in AF protocol. Note that the distortion noises generated at the transmitter could contribute as a source of energy harvesting, while the distortion noises generated at the receiver and the noise $n_{b,r}$ does not contribute to energy harvesting, since the power splitting process is done before the down-conversion process (the majority of the HI distortion noises of receiver is assumed to be caused after the down-conversion process).

Thus, we can parameterize the acquired energy at the relay node as

(3) $$\begin{array}{c} E=\eta \rho (P_{1}|h_{1}{|}^{2}\;+\;P_{2}|h_{2}{|}^{2}\;+\;\kappa_{1}^{2}(P_{1}|h_{1}{|}^{2}\;+\;P_{2}|h_{2}{|}^{2})\;+\;\sigma_{r}^{2})T/2 \end{array}$$

where 0≤η≤1 is the energy-conversion coefficient. The level of hardware impairments in the transmitter and receiver, i.e., $\kappa_{1}$ and $\kappa_{2}$, are required within the region $\kappa_{i},i=\left\{1,2\right\}\in \left[0.08,0.175\right]$ in 3GPP LTE [25]. Thus, (3) can be approximated as

(4) $$\begin{array}{c} E=\eta \rho (P_{1}|h_{1}{|}^{2}+P_{2}|h_{2}{|}^{2}+\sigma_{r}^{2})T/2 \end{array}$$

And the signal delivered to the IP circuit can be expressed as

(5) $$\begin{array}{cc} y_{IP} & =\sqrt{1-\rho }\left(h_{1}\sqrt{P_{1}}x_{1}+h_{2}\sqrt{P_{2}}x_{2}\right)\\ & +\sqrt{1-\rho }\left(h_{1}\sqrt{P_{1}}\tau_{tx,1}+h_{2}\sqrt{P_{2}}\tau_{tx,2}\right)+\tau_{rx,r}+n_{r} \end{array}$$

Assume that perfect channel state information is available for all nodes. With different relaying protocol, the received information $y_{IP}$ will undergo different process which is described in the following and simply depict in Figure 1a. We describe the different process in the next.

***Decode-and-Forward Protocol:*** When applying the DF protocol, the relay first decodes $y_{IP}$ to generate the messages $x_{1}$ and $x_{2}$, then applies a network coding function to construct the transmitted signal as $x_{R}=x_{1}⊕x_{2}$, and finally broadcasts it to both source nodes with the harvested energy.

***Amplify-and-Forward Protocol:*** When applying the AF protocol, the relay utilize an amplifier factor *G* to directly amplify $y_{IP}$ as $x_{R}=G\cdot y_{IP}$ without any decoding and then transfer the amplified signal to both source nodes with the harvested energy.

After information processing, the relay exhausts the harvested energy to forward the processed information to both source nodes. With the consideration of channel reciprocal, the received signals at the source node $S_{i},i=1,2,$ is given by

(6) $$\begin{array}{cc} y_{S_{i}} & =h_{i}\sqrt{P_{R}}\left(x_{R}+\tau_{tx,r}\right)+\tau_{rx,i}+n_{i}\\ & =h_{i}\sqrt{P_{R}}x_{R}+h_{i}\sqrt{P_{R}}\tau_{tx,r}+\tau_{rx,i}+n_{i} \end{array}$$

where $P_{R}$ is the relay’s transmit power, which is calculated as $P_{R}=2E/T$, $\tau_{tx,r}∼\mathrm{CN}\left(0,\kappa_{1}^{2}\right),i=1,2$ is the HI caused by the transmitter of *R*, and $\tau_{rx,i}∼\mathrm{CN}(0,\kappa_{2}^{2}|h_{i}{|}^{2}P_{R})$ are the HI caused by the receiver of $S_{i}$, $n_{i}∼\mathrm{CN}\left(0,\sigma_{i}^{2}\right),\;i=1,2,$ is the noise generated at source $S_{i}$. And

(7) $$x_{R}=\left\{\begin{array}{c} x_{1}⊕x_{2},\;\;\mathrm{for}\mathrm{DF}\mathrm{protocol}\\ G\cdot y_{IP},\;\;\;\mathrm{for}\mathrm{AF}\mathrm{protocol} \end{array}\right.$$

where

(8) $$\begin{array}{cc} G & =[\left(\left(1-\rho \right)\left(1+\kappa_{1}^{2}\right)+\kappa_{2}^{2}\right)\left(\right|h_{1}{|}^{2}P_{1}+|h_{2}{|}_{2}^{P}{)+\sigma_{r}^{2}]}^{-\frac{1}{2}}\\ & =[\left(\bar{\rho }+\kappa^{2}-\rho \kappa_{1}^{2}\right)\left(\right|h_{1}{|}^{2}P_{1}+|h_{2}{|}_{2}^{P}{)+\sigma_{r}^{2}]}^{-\frac{1}{2}} \end{array}$$

where $\bar{\rho }=1-\rho$, $\kappa^{2}=\kappa_{1}^{2}+\kappa_{2}^{2}$.

Since the signal power is usually much greater than the noise power, $\sigma_{r}^{2}$ in (4) and (8) will be omitted in the following analysis.

### 2.3. Instantaneous Achievable Sum Rate: DF Relaying

Referring to [26,27], we obtain the capacity region of the MA phase based on (5) as

(9) $$\begin{array}{cc} & C_{\mathrm{MA}}\left(P_{1},P_{2},h_{1},h_{2}\right)\\ = & \{\left(R_{1},R_{2}\right)|R_{1}\leq C\left(Y_{1R}\right),R_{2}\leq C\left(Y_{2R}\right),\\ & \;\;\;\;R_{1}+R_{2}\leq C\left(Y_{\mathrm{MA}}\right)\}, \end{array}$$

where $C\left(x\right)=\frac{1}{2}\log_{2}\left(1+x\right)$, $Y_{iR},i=\left\{1,2\right\}$, are the end-to-end SNDRs from source node $S_{i}$ to relay *R*, $Y_{\mathrm{MA}}$ is the multiple access signal-to-noise-plus-distortion ratio (SNDR), which are presented as follows

(10a) $$\begin{array}{c} Y_{1R}=\frac{\left(1-\rho \right)\gamma_{1}}{\left(\kappa_{1}^{2}\left(1-\rho \right)+\kappa_{2}^{2}\right)\gamma_{1}+1} \end{array}$$

(10b) $$\begin{array}{c} Y_{2R}=\frac{\left(1-\rho \right)\gamma_{2}}{\left(\kappa_{2}^{2}\left(1-\rho \right)+\kappa_{2}^{2}\right)\gamma_{2}+1} \end{array}$$

(10c) $$\begin{array}{c} Y_{\mathrm{MA}}=\frac{\left(1-\rho \right)\left(\gamma_{1}+\gamma_{2}\right)}{\left(\kappa_{1}^{2}\left(1-\rho \right)+\kappa_{2}^{2}\right)\left(\gamma_{1}+\gamma_{2}\right)+1} \end{array}$$

where $\gamma_{1}=\frac{|h_{1}{|}^{2}P_{1}}{\sigma_{r}^{2}}$, $\gamma_{2}=\frac{|h_{2}{|}^{2}P_{2}}{\sigma_{r}^{2}}$.

With substituting (4) and (7) into (6), we can derive the capacity region of the BC phase as follows referring to [26,27].

(11) $$\begin{array}{cc} & C_{\mathrm{BC}}\left(P_{1},P_{2},h_{1},h_{2}\right)\\ = & \left\{\left(R_{1},R_{2}\right)|R_{1}\leq C\left(Y_{R2}\right),R_{2}\leq C\left(Y_{R1}\right)\right\} \end{array}$$

where $Y_{Ri},i=\left\{1,2\right\}$, are the end-to-end SNDRs from relay *R* to source node $S_{i}$ written as

(12a) $$\begin{array}{c} Y_{R2}=\frac{\eta \rho \gamma_{2}\left(\gamma_{1}+\gamma_{2}\right)}{\kappa^{2}\eta \rho \gamma_{2}\left(\gamma_{1}+\gamma_{2}\right)+V_{2}} \end{array}$$

(12b) $$\begin{array}{c} Y_{R1}=\frac{\eta \rho \gamma_{1}\left(\gamma_{1}+\gamma_{2}\right)}{\kappa^{2}\eta \rho \gamma_{1}\left(\gamma_{1}+\gamma_{2}\right)+V_{1}} \end{array}$$

and $V_{1}=\frac{P_{1}}{\sigma_{1}^{2}}$, $V_{2}=\frac{P_{2}}{\sigma_{2}^{2}}$. For the noise components in the source and relay nodes, it is reasonable to assume $\sigma_{1}^{2}=\sigma_{2}^{2}=\sigma_{r}^{2}=\sigma^{2}$, which will be adopted in the following derivation.

The capacity region of a two-way relay network is constrained by both MA and BC transmissions, therefore, the capacity region of this network exploiting the DF protocol can be presented by

(13) $$\begin{array}{cc} R= & C_{\mathrm{MA}}\left(P_{1},P_{2},h_{1},h_{2}\right)\cap C_{\mathrm{BC}}\left(P_{R},h_{1},h_{2}\right)\\ = & \{\left(R_{1},R_{2}\right)|,R_{1}+R_{2}\leq R_{\mathrm{MA}},\\ & \;\;\;0\leq R_{1}\leq I_{1},\;0\leq R_{2}\leq I_{2}\}, \end{array}$$

where $I_{1}\;=\;\min\;\left(\;C\;\left(\;Y_{1R}\;\right),\;C\;\left(\;Y_{R2}\;\right)\;\right)$, $I_{2}\;=\;\min\;\left(\;C\;\left(\;Y_{2R}\;\right),\;C\;\left(\;Y_{R1}\;\right)\;\right)$, and $R_{\mathrm{MA}}=C\left(Y_{\mathrm{MA}}\right)$.

The total achievable sum rate of the network with the DF protocol is expressed as

(14) $$\begin{array}{cc} R_{\mathrm{sum}}^{\mathrm{DF}} & =R_{1}+R_{2}\\ & =\min\left(I_{1}+I_{2},R_{\mathrm{MA}}\right). \end{array}$$

From (10) and (12), note that under the impact of hardware impairments, a continuous increase of the transmit power does not lead to a continuous increase of SNDR and achievable rate performance for DF-TWR scheme.

### 2.4. Instantaneous Achievable Sum Rate: AF Relaying

Since with the AF protocol directly amplify the received original data, its achievable sum rate is dependent on the end-to-end SNDR. By substituting Equations (5) and (8) into Equation (6), the received signal at $S_{i}$ is rewritten as

(15) $$\begin{array}{cc} y_{S_{i}} & =h_{i}\sqrt{P_{R}}G\cdot y_{IP}+h_{i}\sqrt{P_{R}}\tau +n_{i}\\ & =\underset{\mathrm{self}-\mathrm{interference}\mathrm{signal}}{\underset{︸}{h_{i}h_{\bar{i}}\sqrt{\left(1-\rho \right)P_{i}P_{R}}Gx_{i}}}+\underset{\mathrm{required}\mathrm{signal}}{\underset{︸}{h_{\bar{i}}h_{i}\sqrt{\left(1-\rho \right)P_{\bar{i}}P_{R}}Gx_{\bar{i}}}}\\ & +\underset{\mathrm{impairments}\mathrm{distortion}\mathrm{noise}}{\underset{︸}{h_{i}\sqrt{P_{R}}G\left[\left(h_{1}\sqrt{P_{1}}\;+\;h_{2}\sqrt{P_{2}}\right)\tau_{tx}\;+\;\tau_{rx,r}\right]\;+\;h_{i}\sqrt{P_{R}}\tau }}\\ & +\underset{\mathrm{AWGN}}{\underset{︸}{h_{i}\sqrt{P_{R}}Gn_{r}+n_{i}}}, \end{array}$$

where $i,\bar{i}\in \left\{1,2\right\}$ and $i\neq \bar{i}$, $\tau_{tx}∼\mathrm{CN}\left(0,\kappa_{1}^{2}\right)$, $\tau ∼\mathrm{CN}(0,\kappa^{2})$, and $\kappa^{2}=\sqrt{\kappa_{1}^{2}+\kappa_{2}^{2}}$.

With the perfect knowledge of channel gains and assuming channel reciprocity, $S_{i}$ can remove the self-interference signal from $y_{S_{i}}$ through self-cancellation process. Therefore, we can compute some mathematic manipulations, the SNDR at each node is expressed as follows.

(16) $$\begin{array}{c} Y_{1}=\frac{\Xi_{1}\gamma_{1}\gamma_{2}}{\Xi_{2}\gamma_{1}\left(\gamma_{1}+\gamma_{2}\right)+\eta \rho \gamma_{1}+\Xi_{3}V_{1}} \end{array}$$

for $S_{1}$ (i.e., SNR received at $S_{1}$ transmitted by $S_{2}$) and

(17) $$\begin{array}{c} Y_{2}=\frac{\Xi_{1}\gamma_{1}\gamma_{2}}{\Xi_{2}\gamma_{2}\left(\gamma_{1}+\gamma_{2}\right)+\eta \rho \gamma_{2}+\Xi_{3}V_{2}} \end{array}$$

for $S_{2}$ (i.e., SNR received at $S_{2}$ transmitted by $S_{1}$), where $\Xi_{1}=\eta \rho \left(1-\rho \right)$, $\Xi_{2}=\eta \rho \left[\left(\kappa^{2}-\rho \kappa_{1}^{2}\right)\left(1+\kappa^{2}\right)+\kappa^{2}\left(1-\rho \right)\right]$, $\Xi_{3}=\bar{\rho }+\kappa^{2}-\rho \kappa_{1}^{2}$.

Accordingly, the data rate at $S_{i},i=1,2$, is given by $R_{i}=C\left(Y_{i}\right)$, where $C\left(x\right)=\frac{1}{2}\log_{2}\left(1+x\right)$. Furthermore, the total achievable sum rate of the network with the AF protocol can be expressed as

(18) $$\begin{array}{cc} R_{\mathrm{sum}}^{\mathrm{AF}} & =R_{1}+R_{2}\\ & =C\left(Y_{1}\right)+C\left(Y_{2}\right). \end{array}$$

From Equations (16) and (17), note that under the impact of hardware impairments, a continuous increase of the transmit power does not lead to a continuous increase of SNDR and achievable rate performance for AF-TWR scheme as well.

## 3. Ergodic Capacities Analysis

This section provides the derivation of both DF and AF protocols. The capacity expressions will reveal the effects of hardware impairments on EH-TWR. The channel gain are set to be $h_{i}=g_{i}/d_{i}^{m}$, where the magnitude of $g_{i}$ is modeled as independent but non-identically distribute Rayleigh random variables with variance $\lambda_{i}$, $d_{i}$ is the distance between $S_{i}$ and *R*, and *m* is pass loss exponent.

Two lemmas are presented to help the following derivations of ergodic capacities.

**Lemma** **1.** *Since $g_{i}$ obeys Rayleigh distribute, $\gamma_{i}=\frac{h_{i}P_{i}}{\sigma^{2}}=\frac{g_{i}V_{i}}{d_{i}^{m}}$ also obeys Rayleigh distribute as well. The probability distribution function (PDF) of $\gamma_{i}$ is presented as*

(19) $$\begin{array}{c} f_{\gamma_{i}}\left(\gamma \right)=A_{i}e^{-A_{i}\gamma },\;\mathrm{for}\;\gamma >0 \end{array}$$

*where $A_{i}=\frac{d_{i}^{m}}{\lambda_{i}V_{i}}$, $V_{i}=\frac{P_{i}}{\sigma^{2}}$.*

**Proof.**  The proof is straightforward, which is omitted here. ☐

**Lemma** **2.** *Let* Y *be an arbitrary variable. Then*

(20) $$\begin{array}{c} C=E\left\{C\left(Y\right)\right\}=\frac{1}{2\ln2}\int_{0}^{+\infty }\frac{1-F_{Y}\left(t\right)}{1+t}dt \end{array}$$

*where $C\left(Y\right)=\frac{1}{2}\log_{2}\left(1+Y\right)$*, $F_{Y}\left(\cdot \right)$ *is the cumulative distribution function (CDF) of* Y.

**Proof.**  With $C\left(Y\right)=\frac{1}{2}\log_{2}\left(1+Y\right)$, E{C(Y)} can be obtained by $E\left\{C\left(Y\right)\right\}=\frac{1}{2}\int_{0}^{\infty }\log_{2}\left(1+t\right)f_{Y}\left(t\right)dt$, Then utilizing partial integration, which leads to (20) by using the integration by parts. ☐

### 3.1. Ergodic Capacity of DF Relaying

In order to determine the ergodic capacity of the DF protocol for such an EH-TWR networks, we need to evaluate the ergodic capacity of the source-to-relay link $C_{ir}$, the relay-to-destination link $C_{ri}$, and the multiple-access constraint capacity $C_{\mathrm{mac}}$. Based on Lemma 2, it is non-trivial to obtain the CDFs of each SNDRs: $F_{Y_{iR}}$, $F_{Y_{Ri}}$, and $F_{Y_{\mathrm{MA}}}$ first.

**Theorem** **1.** *Denote $X=\gamma_{1}$, $Y=\gamma_{2}$, the CDFs of $Y_{iR}$, $Y_{Ri}$, and $Y_{\mathrm{MA}}$ is given by*

(21) $$\begin{array}{c} F_{Y_{iR}}\left(t\right)\;=\;1-e^{-\frac{tA_{i}}{\left(1\;-\;\rho \right)\;-\;t(\left(1\;-\;\rho \right)\kappa_{1}^{2}\;+\;t\kappa_{2}^{2}}},\;\;\;0\;<\;t\;<\;\frac{1-\rho }{\left(1\;-\;\rho \right)\kappa_{1}^{2}\;+\;\kappa_{2}^{2}} \end{array}$$

(22) $$\begin{array}{c} F_{Y_{Ri}}\left(t\right)\;=\;1-e^{-A_{i}\sqrt{\frac{tV_{i}}{(1-t\kappa^{2})\eta \rho }}}-A_{\bar{i}}\zeta_{i},\;\;\;\;\;\;0\;<\;t\;<\;\frac{1}{\kappa^{2}} \end{array}$$

*For $F_{Y_{\mathrm{MA}}}\left(t\right)$, there are two cases based on the relative values of $A_{1}$ and $A_{2}$. When $A_{1}=A_{2}$, $0\;<\;t\;<\;\frac{1-\rho }{\left(1-\rho \right)\kappa_{1}^{2}+\kappa_{2}^{2}}$*

(23) $$\begin{array}{c} F_{Y_{\mathrm{MA}}}\;\left(t\right)\;=\;1\;-\;e^{-\frac{tA_{2}}{\bar{\rho }-t\left(\kappa^{2}-\rho \kappa_{1}^{2}\right)}}\;-\;\frac{tA_{2}}{\bar{\rho }-t\left(\kappa^{2}-\rho \kappa_{1}^{2}\right)}e^{-\frac{tA_{1}}{\bar{\rho }-t\left(\kappa^{2}-\rho \kappa_{1}^{2}\right)}}. \end{array}$$

*When $A_{1}\neq A_{2}$, $0\;<\;t\;<\;\frac{1-\rho }{\left(1-\rho \right)\kappa_{1}^{2}+\kappa_{2}^{2}}$*

(24) $$\begin{array}{c} F_{Y_{\mathrm{MA}}}\;\left(t\right)\;=\;1\;+\;\frac{A_{1}}{A_{2}\;\;-\;\;A_{1}}e^{-\frac{tA_{2}}{\bar{\rho }-t\left(\kappa^{2}-\rho \kappa_{1}^{2}\right)}}\;-\;\frac{A_{2}}{A_{2}\;\;-\;\;A_{1}}e^{-\frac{tA_{1}}{\bar{\rho }-t\left(\kappa^{2}-\rho \kappa_{1}^{2}\right)}} \end{array}$$

*where*

(25) $$\begin{array}{c} \zeta_{i}=\int_{0}^{\sqrt{\frac{tV_{i}}{(1-t\kappa^{2})\eta \rho }}}e^{-\left(\left(A_{1}-A_{2}\right)x+\frac{tA_{\bar{i}}V_{i}}{(1-t\kappa^{2})\eta \rho }\cdot \frac{1}{x}\right)}dx \end{array}$$

*and $i,\bar{i}\in \left\{1,2\right\},i\neq \bar{i}$, $\bar{\rho }=1-\rho$.*

**Proof.**  The proof is given in Appendix A. ☐

With the derived CDFs: $F_{Y_{iR}}$, $F_{Y_{Ri}}$, and $F_{Y_{\mathrm{MA}}}$, we can further derive their ergodic capacities as shown in the following Theorem 2 based on Lemma 2.

**Theorem** **2.** *The ergodic capacities $C_{iR}=E\left\{C\left(Y_{iR}\right)\right\}$, $C_{Ri}=E\left\{C\left(Y_{Ri}\right)\right\}$, $C_{\mathrm{MA}}=E\left\{R_{\mathrm{MA}}\right\}$ can be calculated as*

(26) $$\begin{array}{cc} C_{iR}= & \frac{1}{2\;\ln\;2}\;\left[\frac{J_{2}}{1+u}e^{-\;\frac{A_{i}}{\bar{\rho }\;+\;\kappa^{2}\;-\;\rho \kappa_{1}^{2}}}E_{1}\;\left(\frac{A_{i}}{\bar{\rho }\;+\;\kappa^{2}\;-\;\rho \kappa_{1}^{2}}\right)\right.\\ & \left.+J_{1}e^{-\;\frac{A_{i}}{\kappa^{2}\;-\;\rho \kappa_{1}^{2}}}E_{1}\;\left(\frac{A_{i}}{\kappa^{2}\;-\;\rho \kappa_{1}^{2}}\right)\right] \end{array}$$

(27) $$\begin{array}{c} C_{Ri}\;=\;\frac{1}{2\;\ln\;2}\left\{-\frac{2J_{3}}{\kappa^{2}+1}\left[ci\;\;\left(\sqrt{\frac{\Lambda_{i}}{\left(\kappa^{2}+1\right)}}\right)\;\cos\;\;\left(\sqrt{\frac{\Lambda_{i}}{\left(\kappa^{2}+1\right)}}\right)\right.\right.\\ \left.\;+si\;\;\left(\;\;\sqrt{\frac{\Lambda_{i}}{\left(\kappa^{2}\;+\;1\right)}}\right)\;\;\sin\;\;\left(\;\;\sqrt{\frac{\Lambda_{i}}{\left(\kappa^{2}\;+\;1\right)}}\right)\right]\;\;-\;\;2J_{1}\;\;\left[ci\;\;\left(\;\;\sqrt{\frac{\Lambda_{i}}{\kappa^{2}}}\right)\;\;\cos\;\;\left(\;\;\sqrt{\frac{\Lambda_{i}}{\kappa^{2}}}\right)\right.\\ \left.\left.\;+si\;\;\left(\;\;\sqrt{\frac{\Lambda_{i}}{\kappa^{2}}}\right)\;\;\sin\;\;\left(\;\;\sqrt{\frac{\Lambda_{i}}{\kappa^{2}}}\right)\right]\;\;+\;\;\int_{0}^{\;\infty }\;\;\;\;\left(\frac{J_{1}A_{i}}{1\;+\;z}\;+\;\frac{J_{3}A_{i}}{\kappa^{2}\;+\;\left(\kappa^{2}\;+\;1\right)z}\right)\;{\hat{\zeta }}_{i}dz\right\} \end{array}$$

*For $C_{\mathrm{MA}}$, it has are two cases determined by the size of $A_{1}$, $A_{2}$. And for $A_{1}=A_{2}$, $C_{\mathrm{MA}}$ is expressed as*

(28) $$\begin{array}{c} C_{\mathrm{MA}}\;=\;\frac{1}{2\;\ln\;2}\left\{\frac{J_{1}A_{2}}{\kappa^{2}\;-\;\rho \kappa_{1}^{2}}\;\left[\frac{\kappa^{2}\;-\;\rho \kappa_{1}^{2}}{A_{1}}\;-\;e^{\frac{A_{1}}{\kappa^{2}\;-\;\rho \kappa_{1}^{2}}}E_{1}\;\;\left(\frac{A_{1}}{\kappa^{2}\;-\;\rho \kappa_{1}^{2}}\right)\;\right]\right.\\ \;+\;\frac{\left(1\;-\;\rho \right)J_{2}A_{2}}{\kappa^{2}\;-\;\rho \kappa_{1}^{2}}\;\left[\;\frac{1}{A_{1}}\;-\;\frac{e^{\frac{A_{1}}{\left(1\;-\;\rho \right)\;+\;\kappa^{2}\;-\;\rho \kappa_{1}^{2}}}}{\left(1\;\;-\;\;\rho \right)\;\;+\;\;\kappa^{2}\;\;-\;\;\rho \kappa_{1}^{2}}E_{1}\;\;\left(\frac{A_{1}}{\left(1\;\;-\;\;\rho \right)\;\;+\;\;\kappa^{2}\;\;-\;\;\rho \kappa_{1}^{2}}\right)\;\right]\\ \;+\;\frac{J_{2}}{1+\delta_{1}}e^{\frac{A_{2}}{\left(1\;-\;\rho \right)\;+\;\kappa^{2}\;-\;\rho \kappa_{1}^{2}}}\;E_{1}\;\;\left(\frac{A_{2}}{\left(1\;-\;\rho \right)\;+\;\kappa^{2}\;-\;\rho \kappa_{1}^{2}}\right)\\ \left.\;+\;J_{1}e^{\frac{A_{2}}{\kappa^{2}\;-\;\rho \kappa_{1}^{2}}}\;E_{1}\;\;\left(\frac{A_{2}}{\kappa^{2}\;-\;\rho \kappa_{1}^{2}}\right)\right\}. \end{array}$$

*and for $A_{1}\neq A_{2}$, $C_{\mathrm{MA}}$ is expressed as*

(29) $$\begin{array}{c} C_{\mathrm{MA}}=\frac{{\left(A_{2}\;-\;A_{1}\right)}^{-1}}{2\;\ln\;2}\;\left\{\frac{J_{2}A_{1}}{1+\delta_{1}}e^{\frac{A_{2}}{\bar{\rho }+\kappa^{2}-\rho \kappa_{1}^{2}}}E_{1}\;\;\left(\frac{A_{2}}{\bar{\rho }+\kappa^{2}-\rho \kappa_{1}^{2}}\right)\right.\\ \;+\;\frac{J_{2}A_{2}}{1\;+\;\delta_{1}}e^{\frac{A_{1}}{\bar{\rho }\;+\;\kappa^{2}\;-\;\rho \kappa_{1}^{2}}}\;E_{1}\;\;\left(\;\frac{A_{1}}{\bar{\rho }\;+\;\kappa^{2}\;-\;\rho \kappa_{1}^{2}}\;\right)\;\;+\;\;J_{1}A_{2}e^{\frac{A_{1}}{\kappa^{2}\;-\;\rho \kappa_{1}^{2}}}\;E_{1}\;\;\left(\;\frac{A_{1}}{\kappa^{2}\;-\;\rho \kappa_{1}^{2}}\;\right)\\ \left.+J_{1}A_{1}e^{\frac{A_{2}}{\kappa^{2}\;-\;\rho \kappa_{1}^{2}}}E_{1}\left(\frac{A_{2}}{\kappa^{2}\;-\;\rho \kappa_{1}^{2}}\right)\right\} \end{array}$$

*where*

(30a) $$\begin{array}{c} J_{1}=-1 \end{array}$$

(30b) $$\begin{array}{c} J_{2}=1+\kappa^{2} \end{array}$$

(30c) $$\begin{array}{c} J_{3}=1+u,\;u=\kappa_{1}^{2}+\frac{\kappa_{2}^{2}}{1-\rho } \end{array}$$

*and*

(31a) $$\begin{array}{c} {\hat{\zeta }}_{i}=\int_{0}^{\sqrt{\frac{zV_{i}}{\kappa^{2}\eta \rho }}}e^{-\left(\left(A_{1}-A_{2}\right)x+\frac{zA_{\bar{i}}V_{i}}{\kappa^{2}\eta \rho }\cdot \frac{1}{x}\right)}dx \end{array}$$

(31b) $$\begin{array}{c} E_{1}\left(z\right)=\int_{z}^{\infty }\frac{e^{-z}}{z}dz \end{array}$$

(31c) $$\begin{array}{c} \mathrm{ci}\left(z\right)=-\;\;\int_{z}^{\infty }\;\;\frac{\cos z}{z}dz,\;si\left(z\right)=-\;\;\int_{z}^{\infty }\;\;\frac{\sin z}{z}dz \end{array}$$

(31d) $$\begin{array}{c} \Lambda_{i}=\frac{A_{i}^{2}V_{i}}{\eta \rho }. \end{array}$$

**Proof.**  The proof is given in Appendix B. ☐

Using the obtained ergodic capacities of each link, the total ergodic capacity can be obtained by

(32) $$\begin{array}{cc} C_{e}^{\mathrm{DF}} & =\min\left(E\left\{I_{1}\right\}+E\left\{I_{2}\right\},E\left\{R_{\mathrm{MA}}\right\}\right), \end{array}$$

where $E\left\{I_{1}\right\}=\min\left(E\left\{C\left(Y_{1R}\right)\right\},E\left\{C\left(Y_{R2}\right)\right\}\right)$, $E\left\{I_{2}\right\}=\min\left(E\left\{C\left(Y_{2R}\right)\right\},E\left\{C\left(Y_{R1}\right)\right\}\right)$.

### 3.2. Capacity of AF Relaying

For the AF protocol, the total ergodic capacity can be given by

(33) $$\begin{array}{cc} C_{e}^{\mathrm{AF}} & =E\left\{C\left(Y_{1}\right)\right\}+E\left\{C\left(Y_{2}\right)\right\} \end{array}$$

The CDFs of $Y_{1}$ and $Y_{2}$ is presented as following Theorem 3.

**Theorem** **3.** *With the define of $X=\gamma_{1}$ and $Y=\gamma_{2}$. $F_{Y_{i}}\left(t\right)$ is obtained as*

(34) $$\begin{array}{c} F_{Y_{i}}\left(t\right)=1-A_{i}e^{-\frac{tA_{\bar{i}}\eta \rho }{\left(\Xi_{1}-t\Xi_{2}\right)}}\sqrt{\frac{\beta_{i}}{\chi_{i}}}K_{1}\left(\sqrt{\beta_{i}\chi_{i}}\right),\;\;t<\frac{\Xi_{1}}{\Xi_{2}} \end{array}$$

*where*

(35) $$\begin{array}{c} \beta_{i}=\frac{4A_{\bar{i}}\Xi_{3}V_{i}t}{\Xi_{1}-t\Xi_{2}}\\ \chi_{i}=A_{i}+\frac{t\Xi_{2}A_{\bar{i}}}{\Xi_{1}-t\Xi_{2}} \end{array}$$

*and $K_{1}\left(\cdot \right)$ is the first-order modified Bessel function of the second kind [28].*

**Proof.**  The CDF of $Y_{1}$ for the AF protocol is derived as follows. ☐

(36) $$\begin{array}{cc} F_{Y_{1}}\left(t\right) & =\mathrm{Pr}\left\{Y_{1}<t\right\}\\ & =\mathrm{Pr}\left\{\frac{\Xi_{1}\gamma_{1}\gamma_{2}}{\Xi_{2}\gamma_{1}\left(\gamma_{1}+\gamma_{2}\right)+\eta \rho \gamma_{1}+\Xi_{3}V_{1}}<t\right\}\\ & =\mathrm{Pr}\left\{\Xi_{1}XY<t\left(\Xi_{2}X\left(X+Y\right)\;+\;\eta \rho X\;+\;\Xi_{3}V_{1}\right)\right\}\\ & =\mathrm{Pr}\left\{\left(\Xi_{1}\;-\;t\Xi_{2}\right)XY<t\left(\Xi_{2}X^{2}\;+\;\eta \rho X\;+\;\Xi_{3}V_{1}\right)\right\} \end{array}$$

Thus, when $t>\frac{\Xi_{1}}{\Xi_{2}}$, $F_{Y_{1}}\left(t\right)=1$, and when $t<\frac{\Xi_{1}}{\Xi_{2}}$

(37) $$\begin{array}{c} F_{Y_{1}}\left(t\right)=\mathrm{Pr}\left\{Y\;<\;\frac{t\Xi_{2}}{\Xi_{1}\;-\;t\Xi_{2}}X\;+\;\frac{t\eta \rho }{\left(\Xi_{1}\;-\;t\Xi_{2}\right)}\;+\;\frac{t\Xi_{3}V_{1}}{\left(\Xi_{1}\;-\;t\Xi_{2}\right)}\frac{1}{X}\right\}\\ =\;\;\int_{0}^{\;\infty }\;\;\;f_{X}\left(x\right)F_{Y}\left(\frac{t\Xi_{2}}{\Xi_{1}\;-\;t\Xi_{2}}X\;+\;\frac{t\eta \rho }{\left(\Xi_{1}\;-\;t\Xi_{2}\right)}\;+\;\frac{t\Xi_{3}V_{1}}{\left(\Xi_{1}\;-\;t\Xi_{2}\right)}\frac{1}{X}\right)\mathrm{dx}\\ =\;\;\int_{0}^{\;\infty }\;\;\;A_{1}e^{-A_{1}x}\left(1-e^{-A_{2}\left(\frac{t\Xi_{2}}{\Xi_{1}\;-\;t\Xi_{2}}X\;+\;\frac{t\eta \rho }{\left(\Xi_{1}\;-\;t\Xi_{2}\right)}\;+\;\frac{t\Xi_{3}V_{1}}{\left(\Xi_{1}\;-\;t\Xi_{2}\right)}\frac{1}{X}\right)}\right)\mathrm{dx}\\ =1-A_{1}e^{-\frac{tA_{2}\eta \rho }{\left(\Xi_{1}-t\Xi_{2}\right)}}\;\;\int_{0}^{\infty }\;e^{-\left[\left(A_{1}+\frac{tA_{2}\Xi_{2}}{\Xi_{1}-t\Xi_{2}}\right)+\frac{tA_{2}\Xi_{3}V_{1}}{\left(\Xi_{1}-t\Xi_{2}\right)}\frac{1}{x}\right]}\mathrm{dx}. \end{array}$$

Denote $\beta_{1}=\frac{4tA_{2}\Xi_{3}V_{1}}{\Xi_{1}\left(1-t\Xi_{2}\right)}$ and $\chi_{1}=A_{1}+\frac{tA_{2}\Xi_{2}}{1-t\Xi_{2}}$. $F_{Y_{1}}(t$ can be rewrite as shown in (34) by using the equation $\int_{0}^{\infty }\;\;e^{-\frac{\beta }{4x}-\gamma x}\mathrm{dx}=\sqrt{\frac{\beta }{\gamma }}K_{1}\;\left(\sqrt{\beta \gamma }\right)$ ([26, 3.324, 1]). The CDF of $Y_{2}$ can be derived using the same derivation which is omit here due to page limitation. ☐

Substituting the obtained $F_{Y_{i}}\left(t\right)$ into (20) , which gives in Lemma 2, the ergodic capacities $C_{Y_{i}}$ can be calculated as

(38) $$\begin{array}{c} C_{Y_{i}}\;=\;E\left\{C\left(\gamma_{i}\right)\right\}\;=\;\frac{1}{2\ln2}\int_{0}^{\infty }\frac{A_{i}e^{-\frac{zA_{\bar{i}}\eta \rho }{\Xi_{1}\Xi_{2}}}\sqrt{\frac{{\hat{\beta }}_{i}}{{\hat{\chi }}_{i}}}K_{1}\left(\sqrt{{\hat{\beta }}_{i}{\hat{\chi }}_{i}}\right)}{\Xi_{2}{\left(1+z\right)}^{2}+z\left(1+z\right)}dz \end{array}$$

where $z=\frac{t\Xi_{2}}{\Xi_{1}-t\Xi_{2}}$, ${\hat{\beta }}_{i}=\frac{4A_{\bar{i}}\Xi_{3}V_{i}z}{\Xi_{2}}$, ${\hat{\chi }}_{i}=A_{i}+A_{\bar{i}}z$. Then, substitute (38) into (33) will result in the total ergodic of AF protocol.

## 4. Optimal Power Splitting Design

Note that if more received signal is allocated to harvest energy, a higher available transmission power can be obtained, which may lead to a higher transmission rate, but less signal is left for transmission, which may lead to the decrease of transmission rate, and vise versa. Therefore, optimizing ρ is proposed as a way of improving.

With the obtained ergodic capacity expressions of DF and AF protocols derived in Section 3, the OPS value that maximize the ergodic capacity can be obtained by solving the optimization problem:

(39) $$\begin{array}{cc} \mathrm{OP}0: & \;\;\rho^{0}=\underset{\rho }{\arg}\;\left(\mathrm{maximize}\;C_{e}^{Q}\right)\\ & \;\;s.t.\;\;\;0\leq \rho \leq 1 \end{array}$$

where Q = DF for DF protocol, and Q = AF for AF protocol.

However, due to the complicated integral and Bessel function in each ergodic capacity expression, a closed-form solution of the OPS ratio can hardly be obtained. Instead, we are interested in finding the OPS ratio that aims at maximizing the instantaneous achievable sum rate. This optimization is formulated as

(40) $$\begin{array}{cc} \mathrm{OP}1: & \;\;\rho^{0}=\underset{\rho }{\arg}\;\left(\mathrm{maximize}\;R_{\mathrm{sum}}^{Q}\right)\\ & \;\;s.t.\;\;\;0\leq \rho \leq 1 \end{array}$$

where $R_{\mathrm{sum}}^{Q}$ denotes the achievable sum rate of using the DF (i.e., Q = DF) or AF (i.e., Q = AF) protocol. Next we design the OPS ratios for DF and AF protocols, respectively.

### 4.1. The Optimum PS Design for the DF Protocol

From (40), we first analyze the analytical expression $R_{\mathrm{sum}}^{\mathrm{DF}}$ to determine the OPS ratio. Equation (14) shows that the achievable sum rate $R_{\mathrm{sum}}^{\mathrm{DF}}$ is determined by $I_{1}+I_{2}$ and $R_{\mathrm{MA}}$. Since $\log_{2}\left(x\right)$ is a monotonically increasing function of *x*, we note that $R_{\mathrm{MA}}\left(\rho \right)=C\left(Y_{\mathrm{MA}}\right)$ is a decreasing function of ρ because $Y_{\mathrm{MA}}$ is a decreasing function of ρ. The analytical expression of $I\left(\rho \right)=I_{1}\left(\rho \right)+I_{2}\left(\rho \right)$ is expressed in the following proposition.

**Proposition** **1.** *I(ρ) is a three-segment continuous function, segmented by $\rho_{\min}^{\mathrm{DF}}=\min\left(\rho_{1},\rho_{2}\right)$ and $\rho_{\max}^{\mathrm{DF}}=\max\left(\rho_{1},\rho_{2}\right)$. The optimum I(ρ) exists in the range of $\left[\rho_{\min}^{\mathrm{DF}},\rho_{\max}^{\mathrm{DF}}\right]$, where*

(41a) $$\begin{array}{c} \rho_{1}=\frac{\sqrt{{\left(1+b_{1}\right)}^{2}+4b_{1}\kappa_{2}^{2}\gamma_{1}}-\left(1+b_{1}\right)}{2\kappa_{2}^{2}\gamma_{1}} \end{array}$$

(41b) $$\begin{array}{c} \rho_{2}=\frac{\sqrt{{\left(1+b_{2}\right)}^{2}+4b_{2}\kappa_{2}^{2}\gamma_{2}}-\left(1+b_{2}\right)}{2\kappa_{2}^{2}\gamma_{2}} \end{array}$$

(41c) $$\begin{array}{c} b_{1}=\frac{\gamma_{1}V_{2}}{\eta \gamma_{2}\gamma_{\Sigma }},\;b_{2}=\frac{\gamma_{2}V_{1}}{\eta \gamma_{1}\gamma_{\Sigma }} \end{array}$$

*and I(ρ) is given by*

(42) $$I\left(\rho \right)\;=\;\;\left\{\;\;\begin{array}{c} \;C\left(\;\frac{\eta \rho \gamma_{2}\gamma_{\Sigma }}{\kappa^{2}\eta \rho \gamma_{2}\gamma_{\Sigma }+V_{2}}\;\right)\;+\;C\left(\;\frac{\eta \rho \gamma_{1}\gamma_{\Sigma }}{\kappa^{2}\eta \rho \gamma_{1}\gamma_{\Sigma }+V_{1}}\;\right)\\ \;\;\;\;\;\;\;\;\;\;\;\;\;\;\;\;\;\;\;\;\;\;\;\;\;\;\;\;\;\;\;,0\leq \rho \;<\;\rho_{\min}^{\mathrm{DF}}\\ \;C\left(\frac{\left(1-\rho \right)\gamma_{n}}{\left(\kappa_{1}^{2}\left(1\;-\;\rho \right)\;+\;\kappa_{2}^{2}\right)\gamma_{n}\;+\;1}\right)\;+\;C\left(\frac{\eta \rho \gamma_{n}\gamma_{\Sigma }}{\kappa^{2}\eta \rho \gamma_{n}\gamma_{\Sigma }+V_{n}}\right)\\ \;\;\;\;\;\;\;\;\;\;\;\;\;\;\;\;\;\;\;\;\;\;\;\;\;\;\;\;\;\;\;,\rho_{\min}^{\mathrm{DF}}\leq \rho \leq \rho_{\max}^{\mathrm{DF}}\\ \;C\left(\;\frac{\left(1-\rho \right)\gamma_{1}}{\left(\;\kappa_{1}^{2}\;\left(1\;-\;\rho \right)\;+\;\kappa_{2}^{2}\right)\;\gamma_{1}\;+\;1}\;\right)\;+\;C\left(\;\frac{\left(1-\rho \right)\gamma_{2}}{\left(\;\kappa_{1}^{2}\;\left(1\;-\;\rho \right)\;+\;\kappa_{2}^{2}\right)\;\gamma_{2}\;+\;1}\;\right)\\ \;\;\;\;\;\;\;\;\;\;\;\;\;\;\;\;\;\;\;\;\;\;\;\;\;\;\;\;\;\;\;\;\;\;,\rho_{\max}^{\mathrm{DF}}<\rho \leq 1 \end{array}\right.$$

where $\gamma_{\Sigma }=\gamma_{1}+\gamma_{2}$, and n=1 when $\gamma_{1}<\gamma_{2}$, n=2 when $\gamma_{1}\geq \gamma_{2}$.

**Proof.**  The proof is given in Appendix C. ☐

By analyzing the second-order derivation of I(ρ), it is easy to determine that in the region of ρ∈[0,1], the three sections of I(ρ) are, respectively, an increasing function, a concave function and a decreasing function. Since I(ρ) is continuous, the overall function I(ρ) is a concave function. It is straightforward that I(ρ=0)=I(ρ=1)=0, $R_{\mathrm{MA}}\left(\rho =0\right)\neq 0$ and $R_{\mathrm{MA}}\left(\rho =1\right)=0$. Thus, I(ρ) and $R_{\mathrm{MA}}\left(\rho \right)$ have one and only one intersection within the region of ρ∈[0,1]. The achievable sum rate $R_{\mathrm{sum}}^{\mathrm{DF}}\left(\rho \right)=\min\left(I\left(\rho \right),R_{\mathrm{MA}}\left(\rho \right)\right)$, whose analytical expression can be determined by using the following Proposition 2, is achieved by combining I(ρ) and $R_{\mathrm{MA}}\left(\rho \right)$.

**Proposition** **2.** *Denote the second-segment of I(ρ) as* *$I_{sg}\left(\rho \right)=C\left(\frac{\eta \rho \gamma_{n}\gamma_{\Sigma }}{\kappa^{2}\eta \rho \gamma_{n}\gamma_{\Sigma }+V_{n}}\right)+C\left(\frac{\left(1-\rho \right)\gamma_{n}}{\left(\kappa_{1}^{2}\left(1\;-\;\rho \right)\;+\;\kappa_{2}^{2}\right)\gamma_{n}+1}\right),\rho \in \left[0,1\right]$,* *it can get conclude that $I_{sg}\left(\rho \right)$ and $R_{\mathrm{MA}}\left(\rho \right)$ have one and only one intersection point in the range of $\rho \in [0,\rho_{\max}^{\mathrm{DF}}]$. Let $\rho^{+}$ be the intersection of $I_{sg}\left(\rho \right)$ and $R_{\mathrm{MA}}\left(\rho \right)$, there will be two cases for $R_{\mathrm{sum}}^{\mathrm{DF}}\left(\rho \right)$:*

*Case I: If $\rho^{+}<\rho_{\min}^{\mathrm{DF}}$*

(43) $$R_{\mathrm{sum}}^{\mathrm{DF}}\left(\rho \right)\;\;=\;\left\{\begin{array}{c} \;C\;\left(\;\frac{\eta \rho \gamma_{2}\gamma_{\Sigma }}{\kappa^{2}\eta \rho \gamma_{2}\gamma_{\Sigma }+V_{2}}\;\right)\;+\;C\;\left(\;\frac{\eta \rho \gamma_{1}\gamma_{\Sigma }}{\kappa^{2}\eta \rho \gamma_{1}\gamma_{\Sigma }+V_{1}}\;\right),\\ \;\;\;\;\;\;\;\;\;\;\;\;\;\;\;\;\;\;\;\;\;\;\;\;\;\;\;0\leq \rho \leq \rho^{*}\;\\ R_{\mathrm{MA}}\;=\;C\;\left(\frac{\left(1-\rho \right)\gamma_{\Sigma }}{\left(\kappa_{1}^{2}\left(1\;-\;\rho \right)\;+\;\kappa_{2}^{2}\right)\gamma_{\Sigma }+1}\right),\;\rho^{*}\leq \rho \leq 1 \end{array}\right.$$

*Case II: If $\rho^{+}\geq \rho_{\min}^{\mathrm{DF}}$*

(44) $$R_{\mathrm{sum}}^{\mathrm{DF}}\left(\rho \right)\;=\;\left\{\begin{array}{c} \;C\left(\;\frac{\eta \rho \gamma_{2}\gamma_{\Sigma }}{\kappa^{2}\eta \rho \gamma_{2}\gamma_{\Sigma }+V_{2}}\;\right)\;+\;C\left(\;\frac{\eta \rho \gamma_{1}\gamma_{\Sigma }}{\kappa^{2}\eta \rho \gamma_{1}\gamma_{\Sigma }+V_{1}}\;\right)\\ \;\;\;\;\;\;\;\;\;\;\;\;\;\;\;\;\;\;\;\;\;\;\;\;\;\;\;\;,0\leq \rho \leq \rho_{\min}^{\mathrm{DF}}\\ \;C\;\left(\frac{\left(1-\rho \right)\gamma_{n}}{\left(\kappa^{2}\;-\;\rho \kappa_{1}^{2}\right)\gamma_{n}\;+\;1}\right)\;+\;C\;\left(\frac{\eta \rho \gamma_{n}\gamma_{\Sigma }}{\kappa^{2}\eta \rho \gamma_{n}\gamma_{\Sigma }\;+\;V_{n}}\right)\\ \;\;\;\;\;\;\;\;\;\;\;\;\;\;\;\;\;\;\;\;\;\;\;\;\;\;,\rho_{\min}^{\mathrm{DF}}\leq \rho \leq \rho^{+}\\ R_{\mathrm{MA}}=C\;\left(\frac{\left(1-\rho \right)\gamma_{\Sigma }}{\left(\kappa^{2}\;-\;\rho \kappa_{1}^{2}\right)\gamma_{\Sigma }+1}\right),\rho^{+}\leq \rho \leq \rho_{\max}^{\mathrm{DF}} \end{array}\right.$$

*where $\rho^{+}$ is the intersection of $I_{\mathrm{sg}}\left(\rho \right)$ and $R_{\mathrm{MA}}\left(\rho \right)$, which can be obtained by solving the following Kubischen Polynoms*

(45) $$\begin{array}{cc} B_{1}^{+}\rho^{3} & +B_{2}^{+}\rho^{2}+B_{3}^{+}\rho +B_{4}^{+}=0\\ B_{1}^{+}= & \kappa_{1}^{2}\left(1+\kappa_{1}^{2}\right)\eta \gamma_{n}^{2}\gamma_{\Sigma }^{2}\\ B_{2}^{+}= & -\eta \gamma_{n}\gamma_{\Sigma }[\gamma_{n}\kappa_{1}^{2}+\kappa_{1}^{2}\left(1+\kappa^{2}\right)\gamma_{n}\gamma_{\Sigma }\\ & +\left(1+\kappa_{1}^{2}\right)\left(1+\kappa^{2}\right)\left(\gamma_{n}-\kappa^{2}\gamma_{\bar{n}}\right)]\\ B_{3}^{+}= & \left(1+\kappa^{2}\right)\eta \gamma_{n}\gamma_{\Sigma }\left(1+\left(1+\kappa^{2}\right)\left(\gamma_{n}-\kappa^{2}\gamma_{\bar{n}}\right)\right)\\ & +\left(1+\kappa^{2}\left(1+\kappa_{1}^{2}\right)\right)V_{n}\gamma_{\bar{n}}\\ B_{4}^{+}= & -{\left(1+\kappa^{2}\right)}^{2}V_{n}\gamma_{\bar{n}} \end{array}$$

and $\rho^{*}$ is the intersection of the first segment of I(ρ) and $R_{\mathrm{MA}}\left(\rho \right)$, which can be obtained by solving Kubischen Polynoms as follows

(46) $$\begin{array}{cc} B_{1}^{*}\rho^{3} & +B_{2}^{*}\rho^{2}+B_{3}^{*}\rho +B_{4}^{*}=0\\ B_{1}^{*}= & \eta^{2}\gamma_{\Sigma }^{3}\gamma_{1}\gamma_{2}\left(\kappa^{2}\kappa_{2}^{2}-\kappa_{1}^{2}\left(1+\kappa^{2}\right)\right)\\ B_{2}^{*}= & \eta^{2}\gamma_{\Sigma }^{2}\gamma_{1}\gamma_{2}\left(\left(1+2\kappa^{2}\right)+\kappa^{2}\left(1+\kappa^{2}\right)\gamma_{\Sigma }\right)\\ & +\eta \gamma_{\Sigma }^{2}\kappa_{2}^{2}\left(\gamma_{2}V_{1}+\gamma_{1}V_{2}\right)\\ B_{3}^{*}= & \eta \gamma_{\Sigma }\left(\gamma_{2}V_{1}+\gamma_{1}V_{2}\right)+\gamma_{\Sigma }V_{1}V_{2}\\ B_{4}^{*}= & -\gamma_{\Sigma }V_{1}V_{2} \end{array}$$

**Proof.**  The proof is given in the Appendix D. ☐

From the two cases of the achievable sum rates expressed in (43) and (44), we analyze the OPS ratio $\rho^{0}$ by case studies.

#### 4.1.1. Case I: $\rho^{+}<\rho_{\min}^{\mathrm{DF}}$

From Equation (43), we can calculate that the first segment is an increasing function of ρ and the second segment is a decreasing function of ρ. Since $R_{\mathrm{sum}}^{\mathrm{DF}}\left(\rho \right)$ in a continuous piecewise function, it is easy to get that the optimal achievable sum rate is obtained at $\rho^{*}$. Thus, in this case the optimum power splitting ratio is $\rho^{0}=\rho^{*}$.

#### 4.1.2. Case II: $\rho^{+}\geq \rho_{\min}^{\mathrm{DF}}$

From Proposition 1 and the monotonic of $R_{\mathrm{MA}}\left(\rho \right)$, we get that the maximum achievable sum rate exists in the second segment of (44), i.e., $\rho^{0}\in \left[\rho_{\min}^{\mathrm{DF}},\rho^{+}\right]$. Thus, the OPS value is determined by $I_{sg},\;\rho \in \left[\rho_{\min}^{\mathrm{DF}},\;\rho^{+}\right]$. Because $I_{sg}\left(\rho \right),\rho \in \left[0,1\right]$ is a concave function, there should have three subcases to determine the optimal value by analyzing the location of the extrema of of $I_{sg}\left(\rho \right)$. Denote $\rho^{\mathrm{ex}}$ as the extrema of of $I_{sg}\left(\rho \right)$, it can be obtained by solving the following quadratic equation

(47) $$\begin{array}{c} B_{1}^{\mathrm{ex}}\rho^{2}+B_{2}^{\mathrm{ex}}\rho +B_{3}^{\mathrm{ex}}=0\\ B_{1}^{\mathrm{ex}}\;=\;\eta \gamma_{\Sigma }V_{n}\kappa_{1}^{2}\left(1\;+\;\kappa_{1}^{2}\right)\gamma_{n}^{2}\;-\;\kappa^{2}\left(1\;+\;\kappa^{2}\right)\left(1\;+\;\kappa^{2}\gamma_{n}\right)\eta^{2}\gamma_{n}^{2}\gamma_{\Sigma }\\ B_{2}^{\mathrm{ex}}\;=\;-\;\eta \gamma_{\Sigma }V_{n}\left[\gamma_{n}^{2}\left(\;2\kappa^{2}\left(\;1\;+\;\kappa^{2}\;\right)\;+\;\kappa_{1}^{2}\left(\;1\;+\;2\kappa^{2}\;\right)\;\right)\;+\;2\gamma_{n}\left(\;1\;+\;\kappa_{1}^{2}\;+\;\kappa^{2}\;\right)\right]\\ B_{3}^{\mathrm{ex}}\;=\;\eta \gamma_{\Sigma }V_{n}\left[\kappa^{2}\left(1\;+\;\kappa^{2}\right)\gamma_{n}^{2}\;+\;\left(1\;+\;2\kappa^{2}\right)\gamma_{n}\;+\;1\right]\;-\;\left(1\;+\;\kappa^{2}\gamma_{n}\right)V_{n}^{2} \end{array}$$

Through analysis of the locations of the extreme value $\rho^{\mathrm{ex}}$ of $I_{sg}\left(\rho \right)$, we can get three subcases for Case II.

- Subcase 1: $\rho^{\mathrm{ex}}<\rho_{\min}^{\mathrm{DF}}$
  Since $I_{sg}\left(\rho \right)$ is a decreasing function in the range of $\left[\rho^{\mathrm{ex}},1\right]$, it is easy to obtain that $I_{sg},\rho \in \left[\rho_{\min}^{\mathrm{DF}},\rho^{+}\right]$ is also a decreasing function. Thus in this case, the optimum power splitting ratio is $\rho^{0}=\rho_{\min}^{\mathrm{DF}}$.
- Subcase 2: $\rho^{\mathrm{ex}}\in \left[\rho_{\min}^{\mathrm{DF}},\rho^{+}\right]$
  In this case, $I_{sg}\left(\rho \right)$ is an increasing function in the range of $\rho \in [\rho^{\min},\rho^{\mathrm{ex}}]$ and is a decreasing function in the range of $\rho \in [\rho^{\mathrm{ex}},\rho^{+}]$. Thus, $I_{sg},\rho \in \left[\rho_{\min}^{\mathrm{DF}},\rho^{+}\right]$ is a concave function and obtain its maximum at $\rho^{0}=\rho^{\mathrm{ex}}$.
- Subcase 3: $\rho^{\mathrm{ex}}>\rho^{+}$
  $I_{sg}\left(\rho \right)$ is an increasing function in the range of $\left[0,\rho^{\mathrm{ex}}\right]$. So in this case $I_{sg},\rho \in \left[\rho_{\min}^{\mathrm{DF}},\rho^{+}\right]$ is an increasing function of ρ. Its optimum is laid on the border $\rho^{0}=\rho^{+}$.

With the aforementioned analysis, we can obtain the optimal power splitting ratio for DF protocol, which is expressed as follows

(48) $$\rho^{0}=\left\{\begin{array}{c} \rho^{*},\;\;\;\;\;\mathrm{for}\;\rho^{+}<\rho_{\min}^{\mathrm{DF}}\\ \rho_{\min}^{\mathrm{DF}},\;\;\mathrm{for}\;\rho^{+}\geq \rho_{\min}^{\mathrm{DF}}\;\mathrm{and}\;\rho^{\mathrm{ex}}<\rho_{\min}^{\mathrm{DF}}\\ \rho^{\mathrm{ex}},\;\;\;\;\mathrm{for}\;\rho^{+}\geq \rho_{\min}^{\mathrm{DF}}\;\mathrm{and}\;\rho_{\min}^{\mathrm{DF}}\leq \rho^{\mathrm{ex}}<\rho^{+}\\ \rho^{+},\;\;\;\;\mathrm{for}\;\rho^{+}\geq \rho_{\min}^{\mathrm{DF}}\;\mathrm{and}\;\rho^{\mathrm{ex}}>\rho^{+} \end{array}\right.$$

### 4.2. The Optimum PS Design of AF Protocol

Based on (16) and (17), we analyze the OPS ratio for the AF protocol starting from (16) and (17). With the notice of that $Y_{1}$ and $Y_{2}$ are both concave functions of ρ, it is easy to find that $R_{\mathrm{sum}}^{\mathrm{AF}}$ is a concave function. The OPS of AF protocol can be obtained by equating $\frac{\partial R_{\mathrm{sum}}^{\mathrm{AF}}}{\partial \rho }=0$. However, it is difficult to directly calculate a closed form solution of the OPS ratio by solving $\frac{\partial R_{\mathrm{sum}}^{\mathrm{AF}}}{\partial \rho }=0$ because of complex high-order polynomial function of ρ in the numerator and denominator of $\frac{\partial R_{\mathrm{sum}}^{\mathrm{AF}}}{\partial \rho }$. Therefore, we turn to utilize an approximate optimal solution for AF protocol with the consideration of high SNR regimes.

Using the fact that $\log_{2}\left(1+x\right)\approx \log_{2}\left(x\right)$ when x≫1, the instantaneous achievable rate of $S_{i}$ can be simply approximated in high SNR regimes as

(49) $$\begin{array}{c} R_{i}\approx \frac{1}{2}\log_{2}\left(\frac{\Xi_{1}\gamma_{1}\gamma_{2}}{\Xi_{2}\gamma_{\Sigma }\gamma_{i}+\eta \rho \gamma_{i}+\Xi_{3}V_{i}}\right) \end{array}$$

Since the convexity of $\log_{2}\left(x\right)$ is the same with that of *x*, by analyzing the convexity of $\frac{\Xi_{1}\gamma_{1}\gamma_{2}}{\Xi_{2}\gamma_{\Sigma }\gamma_{i}+\eta \rho \gamma_{i}+\Xi_{3}V_{i}}$, it is easy to get that $R_{i}$ is concave function. And, thus, $R_{\mathrm{sum}}^{\mathrm{AF}}\left(\rho \right)=R_{1}+R_{2}$ is concave function.

The first derivatives of $R_{\mathrm{sum}}^{\mathrm{AF}}$ can be obtained as

(50) $$\begin{array}{c} \frac{\partial R_{\mathrm{sum}}^{\mathrm{AF}}}{\partial \rho }=\frac{\partial R_{1}}{\partial \rho }+\frac{\partial R_{2}}{\partial \rho }. \end{array}$$

The obtained $\frac{\partial R_{i}}{\partial \rho }$ is expressed on the top of next page.

(51) $$\begin{array}{c} \frac{\partial R_{i}}{\partial \rho }=\frac{1}{2\ln2}\frac{\left(\eta \gamma_{\Sigma }\gamma_{i}\left(1+\kappa^{2}\right)\kappa_{2}^{2}+\eta \gamma_{i}-\left(1+\kappa_{1}^{2}\right)V_{i}\right)\rho^{2}+2\left(1+\kappa^{2}\right)V_{i}\rho -\left(1+\kappa^{2}\right)V_{i}}{\eta \rho \left(1-\rho \right)\gamma_{1}\gamma_{2}\left[\eta \left(\kappa_{1}^{2}+\kappa_{1}^{2}\kappa^{2}+\kappa^{2}\right)\gamma_{\Sigma }\gamma_{i}\rho^{2}-\left(\eta \kappa^{2}\left(2+\kappa^{2}\right)\gamma_{\Sigma }\gamma_{i}+\eta \gamma_{i}-\left(1+\kappa_{1}^{2}\right)V_{i}\right)\rho -\left(1+\kappa^{2}\right)V_{i}\right]} \end{array}$$

By equating $\frac{\partial R_{1}}{\partial \rho }+\frac{\partial R_{2}}{\partial \rho }$ to zero, we obtain that the OPS ratio that maximizes $R_{\mathrm{sum}}^{\mathrm{AF}}\left(\rho \right)$ must satisfy the following equation:

(52) $$\begin{array}{c} Q_{1}\rho^{4}+Q_{2}\rho^{3}+Q_{3}\rho^{2}+Q_{4}\rho +Q_{5}=0 \end{array}$$

where $Q_{n}=L_{n,1}-L_{n,2}$, n∈{1,2,⋯,5}. And $L_{n,i}$, i∈{1,2}, is expressed as follows

(53) $$\begin{array}{cc} L_{1,i}= & q_{1}\gamma_{\bar{i}}\left[q_{2}\gamma_{i}+\eta \gamma_{i}-\kappa_{1,+}^{2}V_{i}\right]\\ L_{2,i}= & -\left(q_{2}\gamma_{i}+\eta \gamma_{i}-\kappa_{1,+}^{2}V_{i}\right)\left(q_{3}\gamma_{\bar{i}}+\eta \gamma_{\bar{i}}-\kappa_{1,+}^{2}V_{\bar{i}}\right)\\ & +2q_{1}\kappa_{+}^{2}V_{i}\\ L_{3,i}= & -\left(q_{2}\gamma_{i}+\eta \gamma_{i}-\kappa_{1,+}^{2}V_{i}\right)\kappa_{+}^{2}V_{i}-q_{1}\gamma_{\bar{i}}\kappa_{+}^{2}V_{i}\\ & -2\left(q_{3}\gamma_{\bar{i}}+\eta \gamma_{\bar{i}}-\kappa_{1,+}^{2}V_{\bar{i}}\right)\kappa_{+}^{2}V_{i}\\ L_{4,i}= & -2\kappa_{+}^{2}V_{i}V_{\bar{i}}+\left(q_{3}\gamma_{\bar{i}}+\eta \gamma_{\bar{i}}-\kappa_{1,+}^{2}V_{\bar{i}}\right)\kappa_{+}^{2}V_{i}\\ L_{5,i}= & \kappa_{+}^{2}V_{i}V_{\bar{i}} \end{array}$$

and $q_{1}=\eta \left(\kappa_{1}^{2}+\kappa_{1}^{2}\kappa^{2}+\kappa^{2}\right)\gamma_{\Sigma }$, $q_{2}=\eta \kappa_{2}^{2}\left(1+\kappa^{2}\right)\gamma_{\Sigma }$, $q_{3}=\eta \kappa^{2}\left(2+\kappa^{2}\right)\gamma_{\Sigma }$, $\kappa_{1,+}^{2}=1+\kappa_{1}^{2}$, $\kappa_{+}^{2}=1+\kappa^{2}$.

The above function is a quartic polynomial on ρ and has at most four real roots. Since $R_{\mathrm{sum}}^{\mathrm{AF}}\left(\rho \right)$ is a concave function, at most one of these roots of $\frac{\partial R_{\mathrm{sum}}^{\mathrm{AF}}}{\partial \rho }=0$ is real and positive.

## 5. Numerical Results

### 5.1. Effects of Various Parameters on Ergodic Capacity

The purpose of this set of simulations is to validate the correctness of the ergodic capacity expression derived and investigate the effect of various parameter settings on the ergodic capacities for that uses the DF protocol or AF protocols. Parameters chosen are: $\kappa_{1}=\kappa_{2}=\kappa_{\mathrm{ave}}$, $\sigma^{2}=10^{-6}$. The channel gains are considered as Rayleign fading with pass loss, where the variances of the channel coefficients satisfy $\lambda_{1}=\lambda_{2}=1$, and the pass loss exponent is set as m=2.7. The transmit power $P_{1}=P_{2}=P_{t}$, which is within the region $P_{t}=\left[0,30\right]$ dBm.

**Figure 2:**
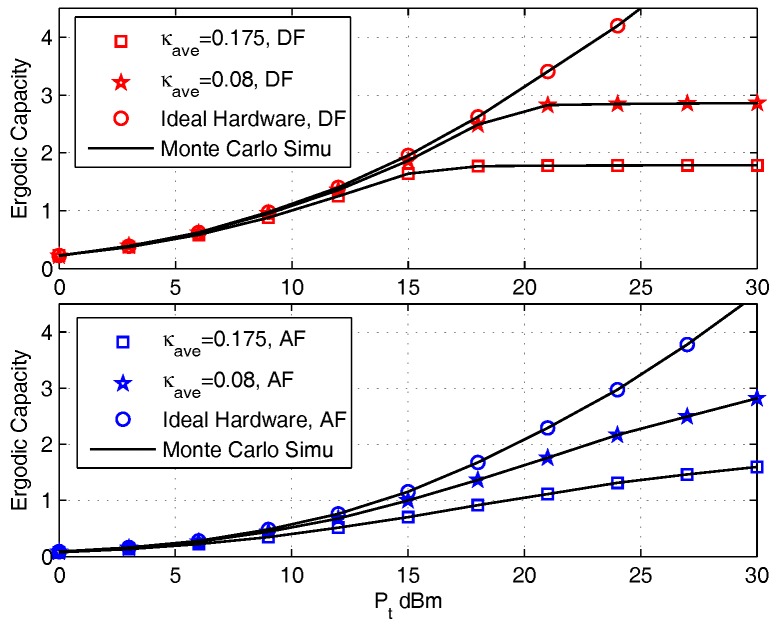
The effect of $\kappa_{\mathrm{ave}}$ on Ergodic Capacity for DF (upper) and AF (lower) Protocols.

Figure 2 shows the effect of $\kappa_{\mathrm{ave}}$ on ergodic capacities. The utilized parameter settings are: ρ=0.5, $d_{1}=d_{2}=5$ m, and η=0.8. The ideal hardware impairment situation $(\kappa_{\mathrm{ave}}=0;$ i.e., no hardware impairments) are presented as benchmark performance. This figure shows that the ergodic capacities obtained by using the closed-form integral expressions are identical with Monte Carlo simulations. When the effects of hardware impairments are considered, the ergodic capacities tend to saturation with the increase of $P_{t}$. And the higher the value of $\kappa_{\mathrm{ave}}$, the more quickly the ergodic capacities saturate. This is because the distortion noises introduced by hardware impairments also increase when $P_{t}$ increases, which will set a bound on the SNDR of each transmission link. Meanwhile, we also find that the effect of $\kappa_{\mathrm{ave}}$ on the DF protocol is more obvious than that on AF protocol. This is because in AF protocol, the amplification of useful signal is more effective than distortion noise with the increase of $P_{t}$, which will slow down the effect degree of the distortion noise, thus do not contribute severe distortion noises than in the DF protocol in the high SNR region.

**Figure 3:**
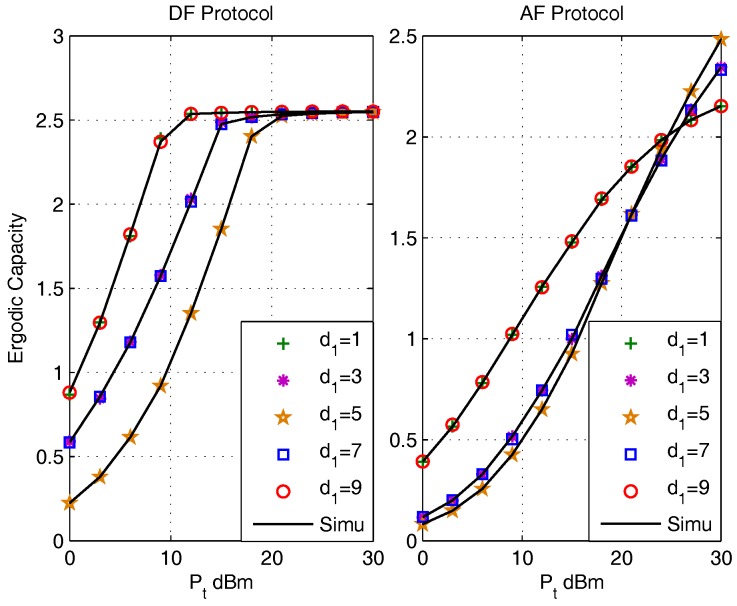
The effect of $d_{1}$ on Ergodic Capacity for DF (left) and AF (right) Protocols.

The effects of relay deployment on ergodic capacities are presented in Figure 3 for the DF protocol (left) and the AF protocol (right). The distance between $S_{1}$ and $S_{2}$ is fixed as $d_{1}+d_{2}=10$ m, where $d_{i},i\in \left\{1,2\right\}$ is the distance between $S_{i}$ and *R*. The utilized parameter settings are: $\kappa_{\mathrm{ave}}=0.1$, ρ=0.5, η=0.8 and destination pair $\left(d_{1},d_{2}\right)=\left\{\left(1,9\right),\left(3,7\right),\left(5,5\right),\left(7,3\right),\left(9,1\right)\right\}$ m. It is observed that the ergodic capacity obtained by the closed-form expressions coincide with that obtained by Monte Carlo simulations (Simu). It is evident that the ergodic capacities when $\left(d_{1},d_{2}\right)=\left\{\left(1,9\right),\left(3,7\right)\right\}$ m are identical with that when $\left(d_{1},d_{2}\right)=\left\{\left(9,1\right),\left(7,3\right)\right\}$ m for both protocols, which infers that the ergodic capacities of both protocols are symmetric with the increase of $d_{1}$. This is because when set $P_{1}=P_{2}$ and fix the distance between $S_{1}$ and $S_{2}$, the system is symmetric, and thus contribute to a symmetric performance. We also find that the DF protocol have the minimum ergodic capacities when the distance pair are set as $\left(d_{1},d_{2}\right)=\left(5,5\right)$ m, the minimum ergodic capacities of AF protocol is obtained when $\left(d_{1},d_{2}\right)=\left(5,5\right)$ m in the low SNR region, and varies to different destination pairs when setting the transmit power $P_{t}$ to some certain values. This abnormal phenomenon of AF is due to the amplification process.

**Figure 4:**
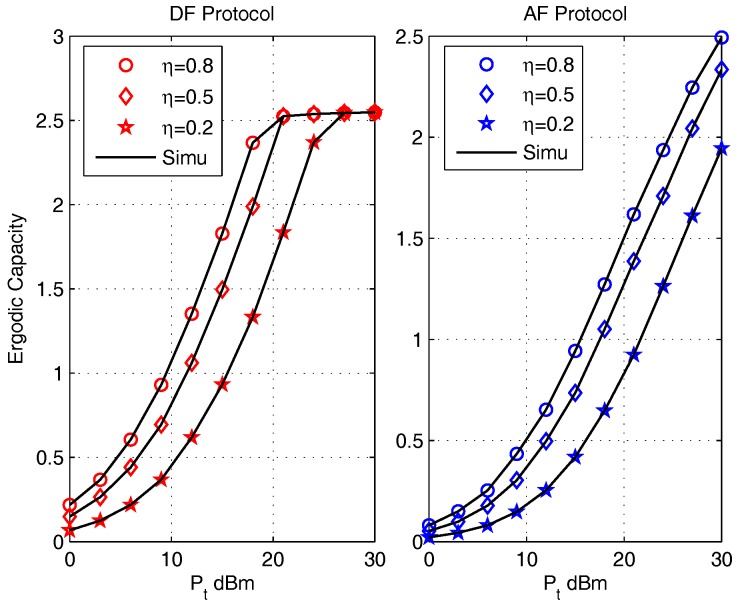
The effect of η on Ergodic Capacity for DF (left) and AF (right) Protocols.

The effect of energy efficient conversion ratio η on ergodic capacities are presented in Figure 4 for the DF protocol (left) and the AF protocol (right) with parameters $\kappa_{\mathrm{ave}}=0.1$, η=0.5, $\left(d_{1},d_{2}\right)=\left(5,5\right)$ m, η=[0.2,0.4,0.6,0.8]. It can be noticed that the ergodic capacities of both protocols obtained by the closed-form expressions are in accordance with that obtained via Monte Carlo simulations (Simu). With the increase of η , it is observed that the ergodic capacity of both protocol increase. This is because $P_{R}$ increases with the increase of η, which will enhance the transmit capabilities of the BC phase. And since $P_{R}$ is provided by energy harvesting, the value of $P_{R}$ is usually not very large. Thus the BC transmission ability dominates the value of ergodic capacity. Thus, with the increase of η, the ergodic capacities increase. The value of η also effects the saturation rate of the DF protocol and the gap between the curves of AF protocol. This phenomenon is due to the value of η effects the value of distortion noises.

**Figure 5:**
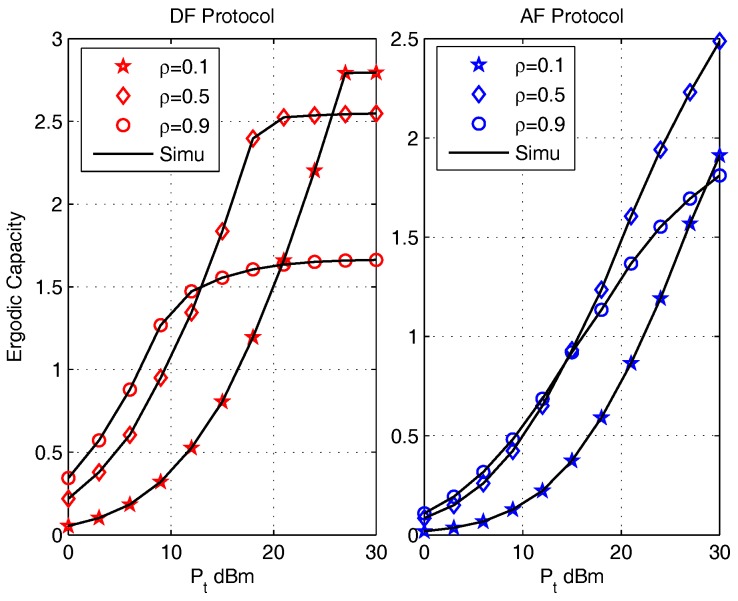
The effect of ρ on Ergodic Capacity for DF (left) and AF (right) Protocols.

The effects of the power splitting ratio ρ on ergodic capacities are evaluated for DF (left) and AF (right) protocols in Figure 5 under the parameters setting: η=0.8, $\left(d_{1},d_{2}\right)=\left(5,5\right)$ m, $\kappa_{\mathrm{ave}}=0.1$, ρ=[0.1,0.5,0.9]. The ergodic capacities of both protocols obtained by the closed-form expressions are in accordance with that obtained via Monte Carlo simulations (Simu). It can be noticed that the maximum ergodic capacities of both DF and AF protocols with ρ=0.9 has best performance in low $P_{t}$ region; while with the increase of $P_{t}$, the maximum ergodic capacity change to the value with parameter setting ρ=0.5 and further change to that with ρ=0.1. This configuration verifies that the ergodic capacities can be enhanced through the design of ρ, which validates the meaning of OPS design analyzed in Section 4.

### 5.2. Effects of Various Parameters on OPS design

The purpose of this set of simulations is to validate the validness of the OPS design and investigate the effect of parameters setting on OPS design for that uses the DF protocol or AF protocol. Since in a practical scenario, the energy conversion efficiency can be known in advance, we mainly focus on the effect of tranmitted power $P_{t}$, impairments distortion level $\kappa_{\mathrm{ave}}$ and relay deployment $d_{1}$ on OPS design in this section. Parameters chosen are: $\kappa_{1}=\kappa_{2}=\kappa_{\mathrm{ave}}$, $\sigma^{2}=10^{-6}$, η=0.8, $d_{1}+d_{2}=10$ m, $\lambda_{1}=\lambda_{2}=1$, m=2.7 and $P_{1}=P_{2}=P_{t}$.

**Figure 6:**
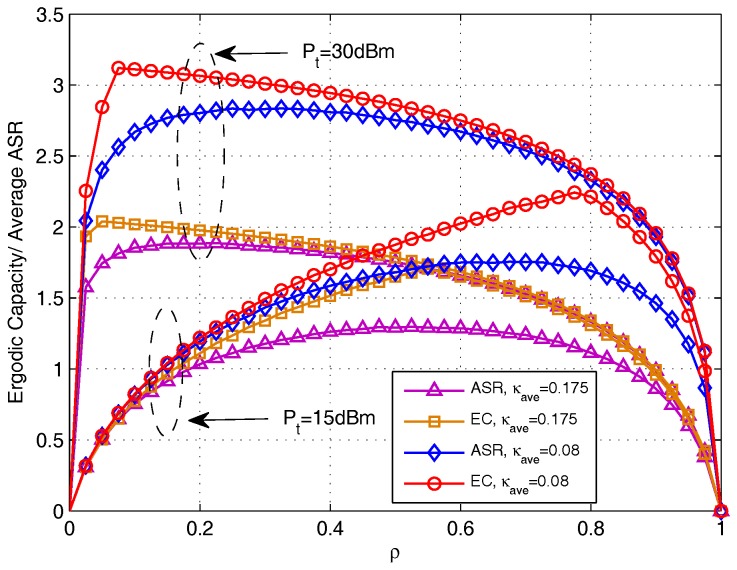
Comparison between ergodic capacity and average achievable sum rate versus ρ for DF protocol.

To better understand the importance and the effect of OPS design, we use ρ as x-axis to see the ergodic capacities (EC) and average achievable sum rate (ASR) changes with the increase of ρ. In Figure 6, we present the effect of PS ratio on EC and on average ASR for DF protocol, where the EC is obtained by using the derived closed-form expressions in Section 3.1 and the average ASR is obtained by doing Monte Carlo simulation using (14). It can be notice that, there exists a maximum value for both ergodic capacity and average ASR with the increase of ρ, and the value of ρ that maximize EC and average ASR varies with different transmit power $P_{t}$ sets and different distortion level $\kappa_{\mathrm{ave}}$ sets. The curves of EC and average ASR are not exactly the same, and with the increase of $P_{t}$ and $\kappa_{\mathrm{ave}}$, the gaps becomes smaller. Though simulation inspect, we find that this is because due to different calculating of EC and average ASR, the correlative influence introduced by multiple-access restraint differs. In addition, with the increase of $P_{t}$ and $\kappa_{\mathrm{ave}}$, the correlative influence comparatively decreases.

**Figure 7:**
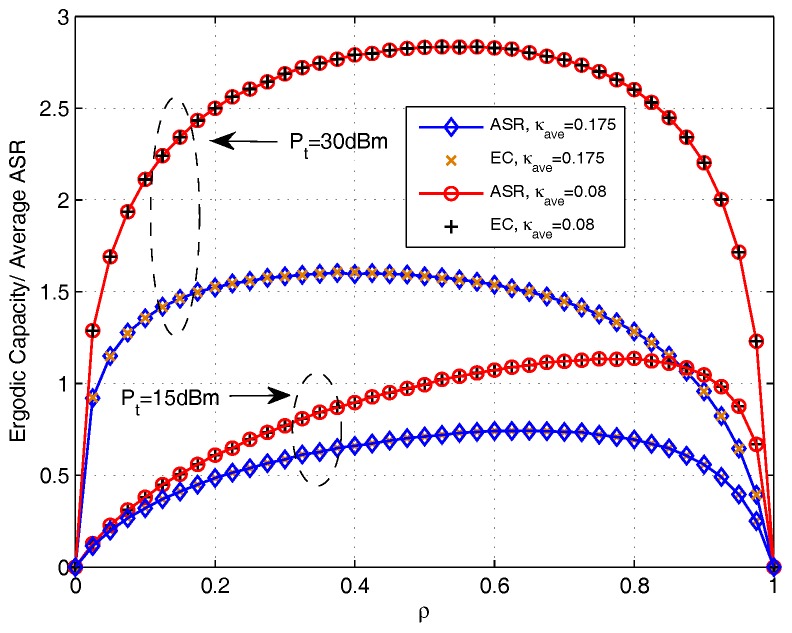
Comparison between ergodic capacity and average achievable sum rate versus ρ for AF protocol.

In Figure 7, the EC and average ASR with the increase of ρ for AF protocol are presented. The EC is obtained by using the derived closed-form expressions in Section 3.2 and the average ASR is obtained by doing Monte Carlo simulation using (18). It can be noted that the curves of EC coincide with the curves of average ASR. This is because without the multiple-access constraint influence in AF protocol, the calculation of EC is equal to the calculation of average ASR. In addition, we also find that the transmit power $P_{t}$ sets and the distortion level $\kappa_{\mathrm{ave}}$ effect the OPS value.

**Figure 8:**
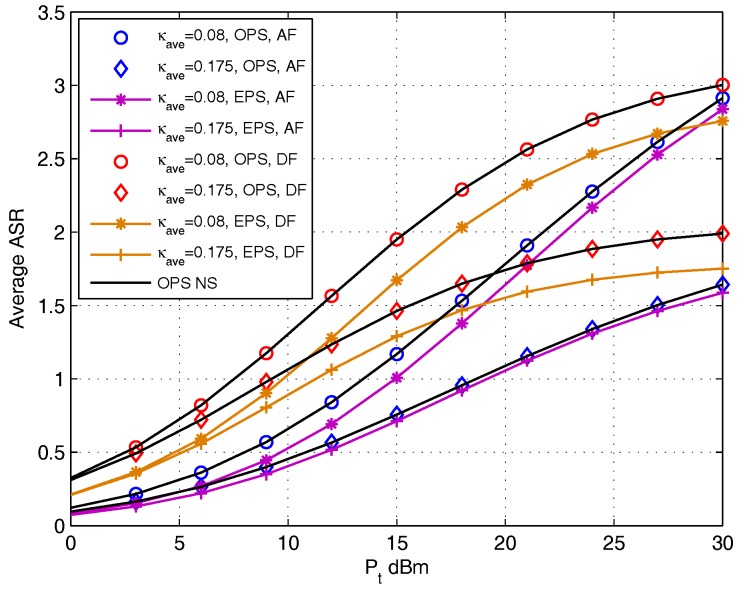
Average achievable sum rate comparison versus $P_{t}$ for DF and AF protocols.

In the following configuration, we analyze the effect of parameters setting on OPS design, and the OPS ratio is obtained aiming at maximizing the instantaneous achievable sum rate which is derived in Section 4. Figure 8 shows the average achievable sum rate as a function of the transmit power $P_{t}$ for DF and AF protocols. The setting of distortion level $\kappa_{\mathrm{ave}}=\left\{0.08,0.175\right\}$ is adopted. Both the numerical search (NS) and the OPS closed-form derived in Section 4 are used to find the OPS ratio and calculate the corresponding achievable sum rate. The achievable sum rate with EPS is presented as benchmark performance. It can be noted that the curves obtained by numerical search coincide with that obtained by using closed-form OPS. It also shows that the OPS design outperforms EPS in each $\kappa_{\mathrm{ave}}$ setting, and the gap grows with the increase of $\kappa_{\mathrm{ave}}$. The effect of OPS design with DF protocol is more obvious than that with AF protocol.

**Figure 9:**
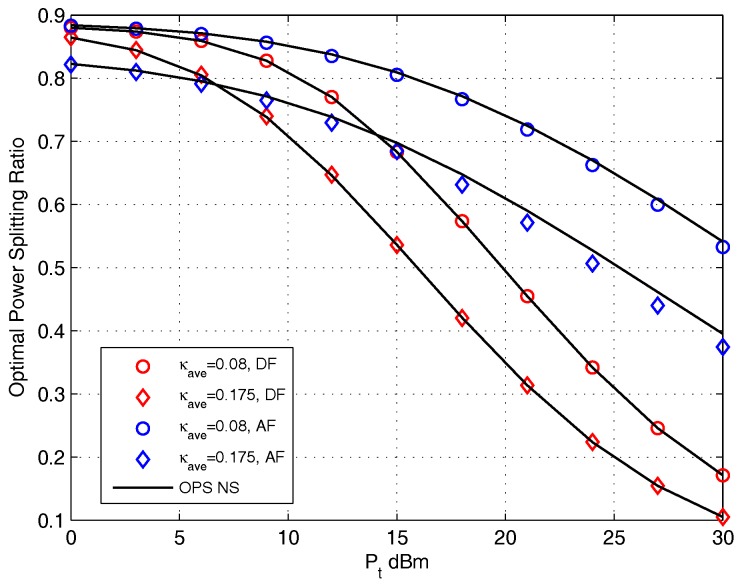
The effect of $P_{t}$ on OPS design for DF and AF protocols.

Figure 9 presents the value of OPS ratio versus the transmit power $P_{t}$ for DF and AF protocols. The setting of distortion level $\kappa_{\mathrm{ave}}=\left\{0.08,0.175\right\}$ is adopted. Seen from this figure, we find that the OPS ratio obtained by closed-form coincides with that obtained by doing NS for DF protocol, and the OPS ratio obtained by closed-form is quite similar with that obtained by doing NS for AF protocol. It is easy to noted that the OPS ratio is a monotonic decreasing function with the increase of $P_{t}$ for both protocols. The reason for this phenomenon is because when the source provide high transmit power, the recruit of harvested energy is more prone to satisfy the relay to destination transmission, and the enhancement of system performance is relies on the source to relay transmission which means that more information needed to be split to information decoder circuit, i.e., (1−ρ) enhances. We also find that the OPS ratio with $\kappa_{\mathrm{ave}}=0.175$ setting is higher than that with $\kappa_{\mathrm{ave}}=0.08$ setting for both protocols. The reason for this phenomenon will be discussed below Figure 11.

**Figure 10:**
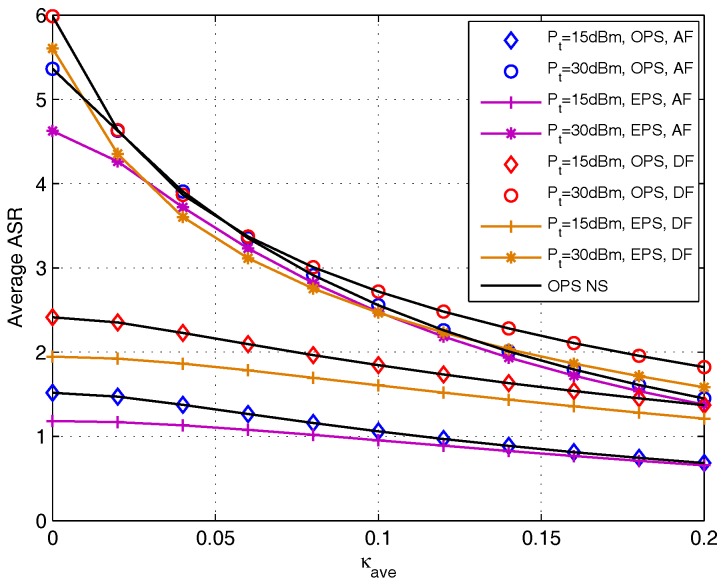
Average achievable sum rate comparison versus $\kappa_{\mathrm{ave}}$ for DF and AF protocols.

Figure 10 plots the achievable sum rate versus hardware impairments distortion level $\kappa_{\mathrm{ave}}$ for both DF and AF protocols with different transmit power $P_{t}=\left\{15,30\right\}$ dBm. The curves with black solid line were generated by utilizing the OPS through numerical search (NS), and the curves with mark were generated by utilizing the OPS derived in Section 4. EPS design is adopted as benchmark performance. As shown in this figure, the achievable sum rate is decreasing and will tend to flatten out as $\kappa_{\mathrm{ave}}$ increases, and the lower the transmit power, the faster the rate approaching to flat. This figure also verifies that OPS design outperforms EPS scheme, and the gaps of DF protocol between OPS and EPS design is bigger than that of AF protocol.

**Figure 11:**
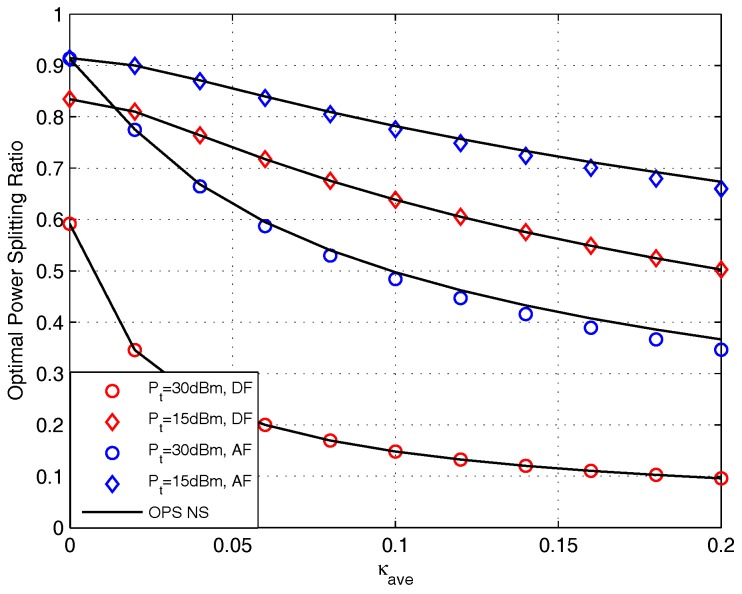
The effect of $\kappa_{\mathrm{ave}}$ on OPS design for DF and AF protocols.

The effect of $\kappa_{\mathrm{ave}}$ on OPS design for DF and AF protocols are presented in Figure 11. This figure presents that the OPS ratio obtained by closed-form is identical with that obtained by doing NS for DF protocol, and for AF protocol, the OPS ratio obtained by closed-form is very close to that obtained by doing NS. It is observed that the OPS ratio is a decreasing function of $\kappa_{\mathrm{ave}}$, and the decreasing slope increases with the increase of $P_{t}$. This is because the higher $\kappa_{\mathrm{ave}}$ makes worse decoding ability. To enhance system performance, more energy need to be diverted to the ID circuit to satisfy the decoding process, thus, OPS value decreases. We also find when $\kappa_{\mathrm{ave}}$ is the same, the OPS value with the higher $P_{t}$ is lower than that with the lower $P_{t}$. This is because in higher $P_{t}$ region, the harvested energy is more prone to satisfy the BC transmission with small ρ.

**Figure 12:**
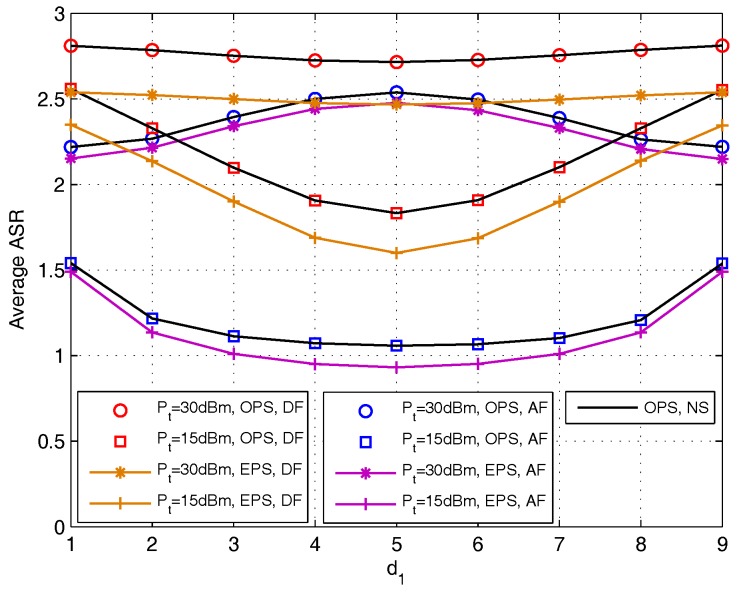
Average achievable sum rate comparison versus $d_{1}$ for DF and AF protocols.

With fixed $\kappa_{\mathrm{ave}}=0.1$, we analyze the effect of relay deployment on average ASR with the set of $P_{t}=\left\{15,30\right\}$ dBm for DF and AF protocols ($d_{1}$ is chosen as the x-axis) in Figure 12. This figure verifies that the average ASR with OPS obtained in Section 4 and that obtained by using NS are the same, and the OPS scheme outperform the EPS scheme. For DF protocol, the average ASR is a convex function with the increase of $d_{1}$ for $P_{t}=\left\{15,30\right\}$ dBm. While for AF protocol, the average ASR is convex function when $P_{t}=15$ dBm, while when $P_{t}=30$ dBm, the average ASR is a concave function. This phenomenon echoes the conclusion in Figure 3 (right) that when $P_{t}$ increase to high enough, the relay is better to deploy close to any one of the source nodes.

**Figure 13:**
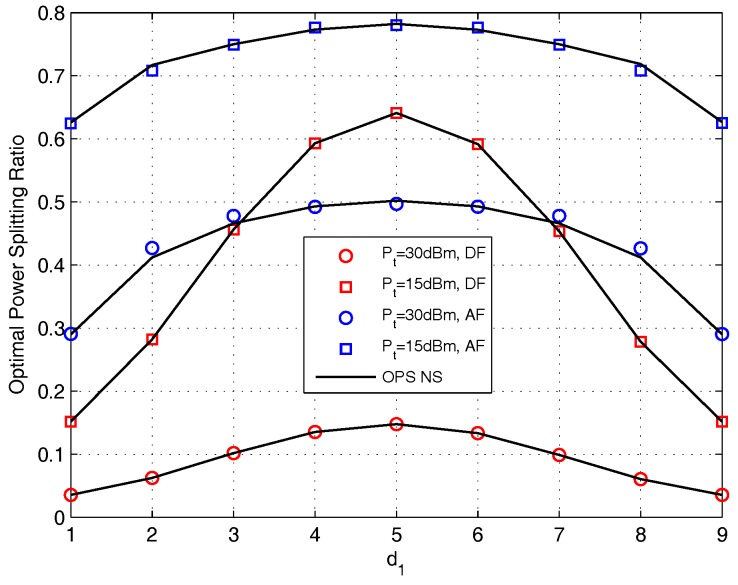
The effect of $d_{1}$ on OPS design for DF and AF protocols.

Figure 13 plots the value of OPS ratio versus $d_{1}$ for DF and AF protocols. It shows that the OPS ratio obtained by closed-form is identical with that obtained by doing NS for DF protocol, and for AF protocol, the OPS ratio obtained by closed-form is very close to that obtained by doing NS. With the increase of $d_{1}$, the OPS values of both DF and AF protocols are concave function. This is because when $P_{1}=P_{2}$ and $d_{1}+d_{2}=10$ m, the system has symmetric nature. In addition, when the relay is deployed in the middle of $S_{1}$ and $S_{2}$, the harvested energy is the smallest with the fixed ρ, and the harvested energy increase when the relay getting closer to any of the source node. Thus, to enhance the performance, more energy is needed to be allocated to energy harvester when $\left(d_{1},d_{2}\right)=\left(5,5\right)$, and when $d_{i},i=1\;\mathrm{or}\;2$ decrease, less energy is needed to be allocate to energy harvester. We also find that the curves with $P_{t}=30$ dBm are lower than that with $P_{t}=15$ dBm. This is because with higher $P_{t}$, the harvested energy is more prone to satisfy the transmission with lower ρ.

## 6. Conclusions

This paper analyzed the a rigorous analysis of a two-way energy harvesting relay network assuming a realistic scenario that the transceiver and receiver hardware is imperfect and will cause distortions. After analyzing two different transmission protocol (DF protocol and AF protocol), we derive the new expression of sum rate with impairment distortions considered. Then, we derive the exact analytical expression of ergodic capacity for both DF and AF relaying protocols. Furthermore, the OPS ratio that maximize the instantaneous achievable sum rate is formulated and solved for both protocols. Through numerical results we find that a continuous increase of the transmit power does not lead to a continuous increase of the ergodic capacity or the achievable sum rate when HI is considered. Also, the effect of HI is more obvious for DF protocol than for AF protocol with the increase of the transmit power. It can be also noticed that the achievable sum-rate with the OPS design outperforms that with EPS design. These results and observations provide very useful insights to guide the system optimization.

## Appendix A

Denote $\gamma_{1}=X$, $\gamma_{2}=Y$, the PDF of *X* and *Y* is $f_{x}\left(x\right)=\int_{0}^{\infty }\;\;A_{1}e^{-A_{1}x}\mathrm{dx},x>0$, $f_{y}\left(y\right)=\int_{0}^{\infty }\;\;A_{2}e^{-A_{2}y}\mathrm{dy},y>0$, respectively, according to Lemma 1.

We first derive the CDF of $Y_{1R}$, which is given by

(A1) $$\begin{array}{cc} F_{Y_{1R}}\left(t\right) & =\mathrm{Pr}\left\{Y_{1R}<t\right\}\\ & =\mathrm{Pr}\left\{\frac{\left(1-\rho \right)\gamma_{1}}{\left(\kappa_{1}^{2}\left(1-\rho \right)+\kappa_{2}^{2}\right)\gamma_{1}+1}<t\right\}\\ & =\mathrm{Pr}\left\{\frac{(1-\rho )X}{(\kappa_{1}^{2}\left(1-\rho \right)+\kappa_{2}^{2})X+1}<t\right\}\\ & =\mathrm{Pr}\left\{[\left(1-\rho \right)\left(1-t\kappa_{1}^{2}\right)+t\kappa_{2}^{2}]X<t\right\} \end{array}$$

From (A1), we note that when $\left(1-\rho \right)\left(1-t\kappa_{1}^{2}\right)+t\kappa_{2}^{2}<0$, $[\left(1-\rho \right)\left(1-t\kappa_{1}^{2}\right)+t\kappa_{2}^{2}]X$ is always less equal than t(t>0), which contribute to $F_{Y_{1R}}\left(t\right)=1$. Whereas, when $\left(1-\rho \right)\left(1-t\kappa_{1}^{2}\right)+t\kappa_{2}^{2}>0$, we have

(A2) $$\begin{array}{cc} F_{Y_{1R}}\left(t\right)= & \mathrm{Pr}\left\{X<\frac{t}{\left(1-\rho \right)\left(1-t\kappa_{1}^{2}\right)+\kappa_{2}^{2}}\right\}\\ = & 1-e^{-\frac{tA_{1}}{\left(1-\rho \right)\left(1-t\kappa_{1}^{2}\right)+\kappa_{2}^{2}}} \end{array}$$

Similar to the derivation of $F_{Y_{1R}}$, we can obtain $F_{Y_{2R}}\left(t\right)=1-e^{-\frac{tA_{2}}{\left(1-\rho \right)\left(1-t\kappa_{1}^{2}\right)+\kappa_{2}^{2}}}$. Thus, $F_{Y_{iR}}$ can be rewrite as shown in (21).

Next, we derive the CDF of $Y_{R1}$, which is express as follows

(A3) $$\begin{array}{cc} F_{Y_{R1}}\left(t\right) & =\mathrm{Pr}\left\{Y_{R1}<t\right\}\\ & =\mathrm{Pr}\left\{\frac{\eta \rho X(X+Y)}{\kappa^{2}\eta \rho X\left(X+Y\right)+V_{1}}<t\right\}\\ & =\mathrm{Pr}\left\{\eta \rho \left(1-t\kappa^{2}\right)X\left(X+Y\right)<tV_{1}\right\} \end{array}$$

It is straightforward that $F_{Y_{R1}}\left(t\right)=1$ when $t>\frac{1}{\kappa^{2}}$. In addition, when $t<\frac{1}{\kappa^{2}}$, $F_{Y_{R1}}\left(t\right)$ can be derived as follows

(A4) $$\begin{array}{c} F_{Y_{R1}}\left(t\right)=\mathrm{Pr}\left\{X\left(X+Y\right)<\frac{tV_{1}}{\eta \rho (1-t\kappa^{2})}\right\}\\ =\mathrm{Pr}\left\{Y<\frac{tV_{1}}{\eta \rho (1-t\kappa^{2})X}-X\right\}\\ =\;\int_{0}^{\;\sqrt{\;\frac{tV_{1}}{\eta \rho (1\;-\;t\kappa^{2})}}}\;\;\;\;\int_{0}^{\frac{tV_{1}}{\eta \rho (1\;-\;t\kappa^{2})}\;\cdot \;\frac{1}{x}\;-\;x}\;A_{1}e^{-A_{1}x}\cdot A_{2}e^{-A_{2}y}\mathrm{dxdy}\\ =\;\int_{0}^{\sqrt{\;\frac{tV_{1}}{\eta \rho (1\;-\;t\kappa^{2})}}}A_{1}e^{-A_{1}x}\left(1-e^{-A_{2}(\frac{tV_{1}}{\eta \rho (1-t\kappa^{2})}\cdot \frac{1}{x}-x)}\right)\mathrm{dx}\\ \;=\;\;1\;\;-\;e^{-\;A_{1}\sqrt{\frac{tV_{1}}{\eta \rho (1\;-\;t\kappa^{2})}}}\;-\;A_{2}\;\;\int_{0}^{\;\sqrt{\frac{tV_{1}}{\eta \rho (1\;-\;t\kappa^{2})}}}e^{-\;\left(A_{1}\;-\;A_{2}\right)x\;+\;\frac{tA_{2}V_{1}}{\eta \rho (1\;-\;t\kappa^{2})}\;\cdot \;\frac{1}{x}}\mathrm{dx} \end{array}$$

Similar to the derivation of $F_{Y_{R1}}$, we can obtain $F_{Y_{R2}}\left(t\right)$. Denote $\zeta_{i}=\int_{0}^{\;\sqrt{\frac{tV_{i}}{\eta \rho (1\;-\;t\kappa^{2})}}}e^{-\;\left(A_{i}\;-\;A_{\bar{i}}\right)x\;+\;\frac{tA_{\bar{i}}V_{i}}{\eta \rho (1\;-\;t\kappa^{2})}\;\cdot \;\frac{1}{x}}dx$, Equation (22) is obtained.

The CDF of $Y_{\mathrm{MA}}$ is derived in the following.

(A5) $$\begin{array}{cc} F_{Y_{\mathrm{MA}}}\left(t\right) & =\mathrm{Pr}\left\{Y_{\mathrm{MA}}<t\right\}\\ & =\mathrm{Pr}\left\{\frac{\left(1-\rho )(X+Y\right)}{(\kappa_{1}^{2}\left(1-\rho \right)+\kappa_{2}^{2})\left(X+Y\right)+1}<t\right\}\\ & =\mathrm{Pr}\left\{\left[\left(1-\rho \right)\left(1-t\kappa_{1}^{2}\right)-t\kappa_{2}^{2}\right]\left(X+Y\right)<t\right\} \end{array}$$

It is straightforward that $F_{Y_{\mathrm{MA}}}\left(t\right)=1$ when $t>\frac{1-\rho }{\left(1-\rho \right)\kappa_{1}^{2}+\kappa_{2}^{2}}$. In addition, when $t<\frac{1-\rho }{\left(1-\rho \right)\kappa_{1}^{2}+\kappa_{2}^{2}}$, $F_{Y_{\mathrm{MA}}}\left(t\right)$ can be derived as follows

(A6) $$\begin{array}{c} F_{Y_{\mathrm{MA}}}\left(t\right)=\mathrm{Pr}\left\{X<\frac{t}{\left(1-\rho \right)\left(1-t\kappa_{1}^{2}\right)-t\kappa_{2}^{2}}-Y\right\}\\ =\mathrm{Pr}\left\{X<\frac{t}{\bar{\rho }-t\kappa^{2}-t\kappa_{1}^{2}\rho }-Y\right\}\\ =\;\int_{0}^{\;\frac{t}{\bar{\rho }-t\kappa^{2}-t\kappa_{1}^{2}\rho }}\;\;\;\;\int_{0}^{\;\frac{t}{\bar{\rho }-t\kappa^{2}-t\kappa_{1}^{2}\rho }\;-\;y}\;A_{1}e^{-A_{1}x}\cdot \;A_{2}e^{-A_{2}y}\mathrm{dxdy}\\ =\;\int_{0}^{\frac{t}{\bar{\rho }-t\kappa^{2}-t\kappa_{1}^{2}\rho }}\;A_{2}e^{-A_{2}y}\left(1-e^{-A_{1}\left(\frac{t}{\bar{\rho }-t\kappa^{2}-t\kappa_{1}^{2}\rho }-y\right)}\right)\mathrm{dy}\\ \;=\;1\;-\;e^{-\frac{tA_{2}}{\bar{\rho }\;-\;t\kappa^{2}\;-\;t\kappa_{1}^{2}\rho }}\;-\;A_{2}e^{-\frac{tA_{1}}{\bar{\rho }\;-\;t\kappa^{2}\;-\;t\kappa_{1}^{2}\rho }}\;\;\;\;\int_{0}^{\frac{t}{\bar{\rho }\;-\;t\kappa^{2}\;-\;t\kappa_{1}^{2}\rho }}e^{-(A_{2}\;-\;A_{1})y}\mathrm{dy} \end{array}$$

Thus, when $A_{1}=A_{2}$,

(A7) $$\begin{array}{c} F_{Y_{\mathrm{MA}}}\left(t\right)=\;1\;-\;e^{-\frac{tA_{2}}{\bar{\rho }\;-\;t\kappa^{2}\;-\;t\kappa_{1}^{2}\rho }}\;-\;\frac{tA_{2}}{\bar{\rho }\;-\;t\kappa^{2}\;-\;t\kappa_{1}^{2}\rho }e^{-\frac{tA_{1}}{\bar{\rho }\;-\;t\kappa^{2}\;-\;t\kappa_{1}^{2}\rho }} \end{array}$$

and when $A_{1}\neq A_{2}$,

(A8) $$\begin{array}{c} F_{Y_{\mathrm{MA}}}\left(t\right)=\;1\;\;-\;e^{\;-\;\frac{tA_{2}}{\bar{\rho }\;-\;t\kappa^{2}\;-\;t\kappa_{1}^{2}\rho }}\;-\;\frac{A_{2}e^{\;-\;\frac{tA_{1}}{\bar{\rho }\;-\;t\kappa^{2}\;-\;t\kappa_{1}^{2}\rho }}}{A_{2}-A_{1}}\left(1\;-\;e^{\;-\;\frac{(A_{2}-A_{1})t}{\bar{\rho }\;-\;t\kappa^{2}\;-\;t\kappa_{1}^{2}\rho }}\right)\\ =1+\frac{A_{1}}{A_{2}-A_{1}}e^{-\frac{tA_{1}}{\bar{\rho }\;-\;t\kappa^{2}\;-\;t\kappa_{1}^{2}\rho }}-\frac{A_{2}}{A_{2}-A_{1}}e^{-\frac{tA_{1}}{\bar{\rho }\;-\;t\kappa^{2}\;-\;t\kappa_{1}^{2}\rho }} \end{array}$$

The above derivations proof the Theorem 1.

## Appendix B

### Appendix B.1. The Derivation of $C_{Ri}$

We first derive $C_{iR}$. Substituting (21) into (20), we obtain

(A9) $$\begin{array}{c} C_{iR}=\frac{1}{2\ln2}\;\int_{0}^{\;\frac{1-\rho }{\left(1-\rho \right)\kappa_{1}^{2}+\kappa_{2}^{2}}}\;\;\frac{e^{-\frac{tA_{i}}{\left(1-\rho \right)-t\left(1-\rho \right)\kappa_{1}^{2}-t\kappa_{2}^{2}}}}{1+t}\mathrm{dt} \end{array}$$

Denote $z=\frac{t(\kappa^{2}-\rho \kappa_{1}^{2})}{\bar{\rho }-t\left(\kappa^{2}-\rho \kappa_{1}^{2}\right)}$, and substitute it into (A9), we obtain

(A10) $$\begin{array}{cc} C_{iR} & =\frac{1}{2\ln2}\;\int_{0}^{+\infty }\frac{e^{-\frac{zA_{i}}{\kappa^{2}-\rho \kappa_{1}^{2}}}}{\left(\kappa_{1}^{2}+\frac{\kappa_{2}^{2}}{1-\rho }\right){\left(1+z\right)}^{2}+z\left(1+z\right)}\mathrm{dz}\\ & =\frac{1}{2\ln2}\;\int_{0}^{+\infty }\frac{e^{-\frac{zA_{i}}{\kappa^{2}-\rho \kappa_{1}^{2}}}}{\left(1+z)(u+(1+u)z\right)}\mathrm{dz}\;\;\;\;\left(\mathrm{where}\;u\;=\;\kappa_{1}^{2}\;+\;\frac{\kappa_{2}^{2}}{1-\rho }\right) \end{array}$$

With some manipulation, it is easy to obtain that $\frac{1}{\left(1+z)(u+(1+u)z\right)}$ can be decomposed into $\frac{J_{1}}{1+z}+\frac{J_{2}}{u+(1+u)z}$, where $J_{1}=-1$, $J_{2}=1+u$. Thus,

(A11) $$\begin{array}{cc} C_{iR}= & \frac{1}{2\ln2}\;\int_{0}^{+\infty }\left(\frac{J_{1}}{1+z}+\frac{J_{2}}{u+(1+u)z}\right)e^{-\frac{zA_{i}}{\kappa^{2}-\rho \kappa_{1}^{2}}}\mathrm{dz}\\ = & \frac{1}{2\ln2}\;\left(\int_{0}^{\infty }\;\;\;\frac{J_{1}e^{-\frac{zA_{i}}{\kappa^{2}-\rho \kappa_{1}^{2}}}}{1\;+\;z}\mathrm{dz}\;+\;\;\int_{0}^{\infty }\;\;\;\frac{J_{2}e^{-\frac{zA_{i}}{\kappa^{2}-\rho \kappa_{1}^{2}}}}{u\;+\;(1\;+\;u)z}\mathrm{dz}\right)\\ = & \frac{1}{2\ln2}\;\left[J_{1}e^{-\;\frac{A_{i}}{\kappa^{2}\;-\;\rho \kappa_{1}^{2}}}E_{1}\;\left(\frac{A_{i}}{\kappa^{2}\;-\;\rho \kappa_{1}^{2}}\right)\right.\\ & \left.+\;\frac{J_{2}}{1\;+\;u}e^{-\;\frac{A_{i}}{\bar{\rho }\;+\;\kappa^{2}\;-\;\rho \kappa_{1}^{2}}}E_{1}\;\left(\frac{A_{i}}{\bar{\rho }\;+\;\kappa^{2}\;-\;\rho \kappa_{1}^{2}}\right)\right] \end{array}$$

Thus (26) is proofed.

### Appendix B.2. The Derivation of $C_{Ri}$

Substituting (22) into (20), we obtain that

(A12) $$\begin{array}{c} C_{Ri}=\frac{1}{2\;\ln\;2}\int_{0}^{\frac{1}{\kappa^{2}}}\;\;\frac{e^{-A_{i}\sqrt{\frac{tV_{i}}{(1-t\kappa^{2})\eta \rho }}}+A_{i}\zeta_{i}}{1+t}\mathrm{dt} \end{array}$$

Denote $z=\frac{t\kappa^{2}}{1-t\kappa^{2}}$, and substitute it into (A12), we obtain

(A13) $$\begin{array}{cc} C_{Ri}= & \frac{1}{2\;\ln\;2}\int_{0}^{\infty }\;\;\frac{e^{-A_{i}\sqrt{\frac{zV_{i}}{\kappa^{2}\eta \rho }}}+A_{i}{\hat{\zeta }}_{i}}{\kappa^{2}{\left(1+z\right)}^{2}+z\left(1+z\right)}\mathrm{dz}\\ = & \frac{1}{2\;\ln\;2}\int_{0}^{\infty }\;\;\frac{e^{-A_{i}\sqrt{\frac{zV_{i}}{\kappa^{2}\eta \rho }}}+A_{i}{\hat{\zeta }}_{i}}{\left(1+z\right)(\kappa^{2}+\left(1+\kappa^{2}\right)z)}\mathrm{dz} \end{array}$$

where ${\hat{\zeta }}_{i}=\int_{0}^{\sqrt{\frac{zV_{i}}{\kappa^{2}\eta \rho }}}e^{-\left(\left(A_{1}-A_{2}\right)x+\frac{zA_{\bar{i}}V_{i}}{\kappa^{2}\eta \rho }\cdot \frac{1}{x}\right)}\mathrm{dx}$

With some manipulation, it is easy to obtain that $\frac{1}{\left(1+z\right)(\kappa^{2}+\left(1+\kappa^{2}\right)z)}=\frac{J_{1}}{1+z}+\frac{J_{3}}{\kappa^{2}+\left(1+\kappa^{2}\right)z}$, where $J_{3}=1+\kappa^{2}$. Thus

(A14) $$\begin{array}{c} C_{Ri}=\frac{1}{2\;\ln\;2}\left[\int_{0}^{\infty }\;\;\frac{J_{1}e^{-A_{i}\sqrt{\frac{zV_{i}}{\kappa^{2}\eta \rho }}}}{1+z}\mathrm{dz}+\int_{0}^{\infty }\;\;\frac{J_{3}e^{-A_{i}\sqrt{\frac{zV_{i}}{\kappa^{2}\eta \rho }}}}{\kappa^{2}+\left(1+\kappa^{2}\right)z}\mathrm{dz}\right.\\ +\left.\int_{0}^{\infty }\;\;\left(\frac{J_{1}}{1+z}+\frac{J_{3}}{\kappa^{2}+\left(1+\kappa^{2}\right)z}\right)A_{i}{\hat{\zeta }}_{i}\mathrm{dz}\right]\\ =\frac{1}{2\;\ln\;2}\;\;\left[2J_{1}\;\;\int_{0}^{\infty }\;\;\;\frac{we^{-A_{1}w}}{\frac{V_{1}}{\kappa^{2}\eta \rho }\;+\;w^{2}}\mathrm{dw}\;+\;\frac{2J_{3}}{1\;+\;\kappa^{2}}\;\;\int_{0}^{\infty }\;\;\;\frac{we^{-A_{i}w}}{\frac{V_{i}}{\kappa_{\;+}^{2}\eta \rho }\;+\;w^{2}}\mathrm{dw}\right.\\ +\left.\int_{0}^{\infty }\;\;\left(\frac{J_{1}}{1+z}+\frac{J_{3}}{\kappa^{2}+\left(1+\kappa^{2}\right)z}\right)A_{i}{\hat{\zeta }}_{i}\mathrm{dz}\right]\;\;\;\;\left(\mathrm{where}\;w=\sqrt{\frac{zV_{i}}{\kappa^{2}\eta \rho }}\right) \end{array}$$

Referring to [28, 3.354, 2], (27) is obtained.

### Appendix B.3. The Derivation of $C_{\mathrm{MAC}}$

(1) When $A_{1}=A_{2}$

Substituting (23) into (20), we obtain

(A15) $$\begin{array}{c} C_{\mathrm{MAC}}=\frac{1}{2\;\ln\;2}\;\int_{0}^{\infty }\;\;\frac{1}{1\;-\;t}\;\;\left[e^{-\frac{tA_{2}}{\bar{\rho }\;-\;t\left(\kappa^{2}\;-\;\rho \kappa_{1}^{2}\right)}}\;-\;\frac{tA_{2}e^{-\frac{tA_{1}}{\bar{\rho }\;-\;t\left(\kappa^{2}\;-\;\rho \kappa_{1}^{2}\right)}}}{\bar{\rho }\;-\;t\left(\kappa^{2}\;-\;\rho \kappa_{1}^{2}\right)}\right]\;\;\mathrm{dt} \end{array}$$

Similar to the derivation of $C_{iR}$, we denote $z=\frac{t(\kappa^{2}-\rho \kappa_{1}^{2})}{\bar{\rho }-t\left(\kappa^{2}-\rho \kappa_{1}^{2}\right)}$, and (A15) can rewrite as

(A16) $$\begin{array}{c} C_{\mathrm{MAC}}\;=\;\frac{1}{2\;\ln\;2}\;\left[\;\underset{\Delta_{1}}{\underset{︸}{\int_{0}^{\infty }\;\;\;\frac{J_{1}}{1\;+\;z}e^{-\frac{zA_{2}}{\kappa^{2}\;-\;\rho \kappa_{1}^{2}}}\mathrm{dz}\;+\;\;\int_{0}^{\infty }\;\;\;\frac{J_{2}e^{-\frac{zA_{2}}{\kappa^{2}\;-\;\rho \kappa_{1}^{2}}}}{u\;+\;(1\;+\;u)z}\mathrm{dz}}}\right.\\ \left.\;+\;\;\underset{\Delta_{2}}{\underset{︸}{\int_{0}^{\infty }\;\;\;\frac{J_{1}}{1\;+\;z}\frac{zA_{2}e^{-\frac{zA_{1}}{\kappa^{2}\;-\;\rho \kappa_{1}^{2}}}}{\kappa^{2}\;-\;\rho \kappa_{1}^{2}}\mathrm{dz}\;+\;\;\;\int_{0}^{\infty }\;\;\;\frac{J_{2}}{u\;+\;(1\;+\;u)z}\frac{zA_{2}e^{-\frac{zA_{1}}{\kappa^{2}\;-\;\rho \kappa_{1}^{2}}}}{\kappa^{2}\;-\;\rho \kappa_{1}^{2}}\mathrm{dz}}}\right] \end{array}$$

The value of $\Delta_{1}$ can be derived following the derivation of $C_{iR}$. In addition, $\Delta_{2}$ can be obtained by using the integral formula presented in [26, 3.353, 5], which is $\int_{0}^{\infty }\;\;\frac{x^{n}e^{-\mu x}}{x+\beta }\mathrm{dx}={\left(-1\right)}^{n}\beta^{n}e^{\eta \mu }E_{1}\left(\eta \mu \right)+\sum_{k=1}^{n}\left(k-1\right)!{\left(-\beta \right)}^{n-k}\mu^{-k}$. Combined $\Delta_{1}$ and $\Delta_{2}$, (28) is obtained.

(2) When $A_{1}\neq A_{2}$

Substituting (24) into (20), we obtain

(A17) $$\begin{array}{c} C_{\mathrm{MAC}}\;=\;\frac{1}{2\;\ln\;2}\;\int_{0}^{\infty }\;\;\;\frac{1}{1\;-\;t}\;\;\left[\frac{A_{2}e^{-\frac{tA_{1}}{\bar{\rho }\;-\;t\left(\kappa^{2}\;-\;\rho \kappa_{1}^{2}\right)}}}{A_{2}\;-\;A_{1}}\;-\;\frac{A_{1}e^{-\frac{tA_{2}}{\bar{\rho }\;-\;t\left(\kappa^{2}\;-\;\rho \kappa_{1}^{2}\right)}}}{A_{2}\;-\;A_{1}}\right]\;\;\mathrm{dt} \end{array}$$

Denote $z=\frac{t(\kappa^{2}-\rho \kappa_{1}^{2})}{\bar{\rho }-t\left(\kappa^{2}-\rho \kappa_{1}^{2}\right)}$, Equation (A17) changes into the following equation with some manipulations.

(A18) $$\begin{array}{c} C_{\mathrm{MAC}}\;=\;\frac{{\left(A_{2}\;\;-\;\;A_{1}\right)}^{-1}}{2\;\ln\;2}\;\;\left[\;J_{1}A_{2}\;\;\int_{0}^{\;\infty }\;\;\;\frac{e^{\;-\;\frac{zA_{1}}{\kappa^{2}\;-\;\rho \kappa_{1}^{2}}}}{1\;+\;z}\mathrm{dz}\;+\;\;J_{1}A_{1}\;\;\int_{0}^{\;\infty }\;\;\;\frac{e^{\;-\;\frac{zA_{2}}{\kappa^{2}\;-\;\rho \kappa_{1}^{2}}}}{1\;+\;z}\mathrm{dz}\right.\\ \left.+J_{2}A_{2}\;\;\int_{0}^{\infty }\;\;\;\frac{e^{-\frac{zA_{1}}{\kappa^{2}\;-\;\rho \kappa_{1}^{2}}}}{u\;+\;(1\;+\;u)z}\mathrm{dz}+J_{2}A_{1}\;\;\int_{0}^{\infty }\;\;\;\frac{e^{-\frac{zA_{2}}{\kappa^{2}\;-\;\rho \kappa_{1}^{2}}}}{u\;+\;(1\;+\;u)z}\mathrm{dz}\right]\\ \;=\;\frac{{\left(A_{2}\;\;-\;\;A_{1}\right)}^{-1}}{2\;\ln\;2}\;\left[J_{1}\;A_{2}e^{\frac{A_{1}}{\kappa^{2}-\rho \kappa_{1}^{2}}}\;E_{1}\;\;\left(\;\frac{A_{1}}{\kappa^{2}\;\;-\;\;\rho \kappa_{1}^{2}}\;\right)\right.\\ +\;J_{1}\;A_{1}e^{\frac{A_{2}}{\kappa^{2}-\rho \kappa_{1}^{2}}}\;E_{1}\;\;\left(\;\frac{A_{2}}{\kappa^{2}\;\;-\;\;\rho \kappa_{1}^{2}}\;\right)\;+\;\frac{J_{2}\;A_{2}}{1\;+\;u}e^{\frac{A_{1}}{\bar{\rho }\;+\;\kappa^{2}\;-\;\rho \kappa_{1}^{2}}}\;E_{1}\;\;\left(\;\frac{A_{1}}{\bar{\rho }\;+\;\;\kappa^{2}\;-\;\;\rho \kappa_{1}^{2}}\;\right)\\ \left.+\frac{J_{2}A_{1}}{1+u}e^{\frac{A_{2}}{\bar{\rho }+\kappa^{2}-\rho \kappa_{1}^{2}}}\;E_{1}\left(\frac{A_{2}}{\bar{\rho }+\kappa^{2}-\rho \kappa_{1}^{2}}\right)\right] \end{array}$$

Thus, (29) is obtained.

## Appendix C

As a simple extension, the two-way relaying system can be treated as two one-way relay systems. We first obtain two suboptimal power splitting designs $\rho_{i},i=1,2,$ which maximize $I_{1}\left(\rho \right)$ or maximize $I_{2}\left(\rho \right)$ to obtain $I_{\mathrm{sub},i}=I\left(\rho_{i}\right)$.

Note that $I_{i},i=1,2$ is determined by $Y_{ir}\left(\rho \right)=C\left(\frac{\left(1-\rho \right)\gamma_{1}}{\left(\kappa_{1}^{2}\left(1-\rho \right)+\kappa_{2}^{2}\right)\gamma_{1}+1}\right)$ and $Y_{r\bar{i}}\left(\rho \right)=C\left(\frac{\eta \rho \gamma_{\bar{i}}\left(\gamma_{1}+\gamma_{2}\right)}{\kappa^{2}\eta \rho \gamma_{\bar{i}}\left(\gamma_{1}+\gamma_{2}\right)+V_{\bar{i}}}\right)$, where $i,\bar{i}=1,2,i\neq \bar{i}$. Through calculation of the second derivation, we find that $Y_{ir}\left(\rho \right)$ is a decreasing functions in the region of ρ∈[0,1], which decreases from a positive real number to 0, while $Y_{r\bar{i}}\left(\rho \right)$ is an increasing function, which increases from 0 to a positive real number. Thus, $Y_{ir}\left(\rho \right)=Y_{r\bar{i}}\left(\rho \right)$ has one and only one solution $\rho_{i}$ (intersection), which is the optimum value since ρ is continuous. This shows that the optimal value $\rho_{i}$ can be obtained can be obtained via (41a,b).

Denote $\rho_{\min}^{\mathrm{DF}}=\min\left(\rho_{1},\rho_{2}\right)$ and $\rho_{\max}^{\mathrm{DF}}=\max\left(\rho_{1},\rho_{2}\right)$. By substituting $\rho_{\min}^{\mathrm{DF}}$ and $\rho_{\max}^{\mathrm{DF}}$ into I(ρ), we obtain two suboptimal values $I_{\mathrm{sub},\min}^{\mathrm{DF}}=I\left(\rho_{\min}^{\mathrm{DF}}\right)$ and $I_{\mathrm{sub},\max}^{\mathrm{DF}}=I\left(\rho_{\max}^{\mathrm{DF}}\right)$. It is easy to notice that $I\left(\rho_{\min}^{\mathrm{DF}}-\Delta \rho \right)<I\left(\rho_{\min}^{\mathrm{DF}}\right)$, because in the range of $\rho \in [0,\rho_{\min}^{\mathrm{DF}}]$ both of $I_{1}\left(\rho \right)$ and $I_{2}\left(\rho \right)$ are increasing functions; and $I\left(\rho_{\max}^{\mathrm{DF}}+\Delta \rho \right)<I\left(\rho_{\max}^{\mathrm{DF}}\right)$, because in the range of $\rho \in [\rho_{\max}^{\mathrm{DF}},1]$ both of $I_{1}\left(\rho \right)$ and $I_{2}\left(\rho \right)$ are decreasing functions, where 0<Δρ<1 and $\left\{\rho_{\min}^{\mathrm{DF}}-\Delta \rho ,\rho_{\max}^{\mathrm{DF}}+\Delta \rho \right\}\in \left[0,1\right]$. In the range of $\left[\rho_{\min}^{\mathrm{DF}},\rho_{\max}^{\mathrm{DF}}\right]$, $I_{1}\left(\rho \right)$ and $I_{2}\left(\rho \right)$ have opposite monotonicity; thus there exist only one intersection point within the range of $\left[\rho_{\min}^{\mathrm{DF}},\rho_{\max}^{\mathrm{DF}}\right]$, which is the maximum value of I(ρ). With the above analysis, we can rewrite I(ρ) as shown in (42).

## Appendix D

As a simple extension, the two-way relaying system can be treated as two one-way relay systems. We first obtain two suboptimal power splitting designs $\rho_{i},i=1,2,$ which maximize $I_{1}\left(\rho \right)$ or maximize $I_{2}\left(\rho \right)$ to obtain $I_{\mathrm{sub},i}=I\left(\rho_{i}\right)$.

Note that $I_{i},i=1,2$ is determined by $Y_{ir}\left(\rho \right)=C\left(\frac{\left(1-\rho \right)\gamma_{1}}{\left(\kappa_{1}^{2}\left(1-\rho \right)+\kappa_{2}^{2}\right)\gamma_{1}+1}\right)$ and $Y_{r\bar{i}}\left(\rho \right)=C\left(\frac{\eta \rho \gamma_{\bar{i}}\left(\gamma_{1}+\gamma_{2}\right)}{\kappa^{2}\eta \rho \gamma_{\bar{i}}\left(\gamma_{1}+\gamma_{2}\right)+V_{\bar{i}}}\right)$, where $i,\bar{i}=1,2,i\neq \bar{i}$. Through calculation of the second derivation, we find that $Y_{ir}\left(\rho \right)$ is a decreasing function in the region of ρ∈[0,1], which decreases from a positive real number to 0, while $Y_{r\bar{i}}\left(\rho \right)$ is an increasing function, which increases from 0 to a positive real number. Thus, $Y_{ir}\left(\rho \right)=Y_{r\bar{i}}\left(\rho \right)$ has one and only one solution $\rho_{i}$ (intersection), which is the optimum value since ρ is continuous. This shows that the optimal value $\rho_{i}$ can be obtained can be obtained via (41a,b).

Denote $\rho_{\min}^{\mathrm{DF}}=\min\left(\rho_{1},\rho_{2}\right)$ and $\rho_{\max}^{\mathrm{DF}}=\max\left(\rho_{1},\rho_{2}\right)$. By substituting $\rho_{\min}^{\mathrm{DF}}$ and $\rho_{\max}^{\mathrm{DF}}$ into I(ρ), we obtain two suboptimal values $I_{\mathrm{sub},\min}^{\mathrm{DF}}=I\left(\rho_{\min}^{\mathrm{DF}}\right)$ and $I_{\mathrm{sub},\max}^{\mathrm{DF}}=I\left(\rho_{\max}^{\mathrm{DF}}\right)$. Notice that $I\left(\rho_{\min}^{\mathrm{DF}}-\Delta \rho \right)<I\left(\rho_{\min}^{\mathrm{DF}}\right)$, because in the range of $\rho \in [0,\rho_{\min}^{\mathrm{DF}}]$ both of $I_{1}\left(\rho \right)$ and $I_{2}\left(\rho \right)$ are increasing functions; and $I\left(\rho_{\max}^{\mathrm{DF}}+\Delta \rho \right)<I\left(\rho_{\max}^{\mathrm{DF}}\right)$, because in the range of $\rho \in [\rho_{\max}^{\mathrm{DF}},1]$ both of $I_{1}\left(\rho \right)$ and $I_{2}\left(\rho \right)$ are decreasing functions, where 0<Δρ<1 and $\left\{\rho_{\min}^{\mathrm{DF}}-\Delta \rho ,\rho_{\max}^{\mathrm{DF}}+\Delta \rho \right\}\in \left[0,1\right]$. In the range of $\left[\rho_{\min}^{\mathrm{DF}},\rho_{\max}^{\mathrm{DF}}\right]$, $I_{1}\left(\rho \right)$ and $I_{2}\left(\rho \right)$ have opposite monotonicity; thus there exist only one intersection point within the range of $\left[\rho_{\min}^{\mathrm{DF}},\rho_{\max}^{\mathrm{DF}}\right]$, which is the maximum value of I(ρ). With the above analysis, we can rewrite I(ρ) as shown in (42).
